# How am I doing? It varies: mixed-effects location scale modeling to examine intra-individual variability in health-related research

**DOI:** 10.1007/s10865-026-00638-6

**Published:** 2026-02-20

**Authors:** Mirinda Whitaker, Akiko Okifuji, Pascal Deboeck

**Affiliations:** 1 Department of Psychology, Ohio University, Athens, OH USA; 2 Department of Anesthesiology, University of Utah, Salt Lake City, UT USA; 3 Department of Psychology, University of Utah, Salt Lake City, UT USA

**Keywords:** Intraindividual standard deviation, Within-individual variance, Intra-individual variability, Multilevel modeling, Mixed-effects location scale modeling

## Abstract

Intra-individual variability in many behavioral and medical constructs, including pain, sleep, cognitive performance, and emotion is of growing interest. However, intraindividual variability is often quantified using the intra-individual standard deviation (iSD), or related measures like variance or the coefficient of variability, all of which are statistically inefficient and unreliable when calculated on relatively few observations. One underused methodological tool for modeling variance is mixed-effects location scale (MELS) modeling. Unlike typical multi-level models, MELS allows for random effects on variance components, alongside random slopes and intercepts, which provides the ability to ask questions about inter-individual differences in intra-individual variability. The present tutorial will introduce the MELS model with an application to data on chronic pain symptoms over time. In this tutorial, we teach the MELS model and demonstrate software through a series of three increasingly sophisticated applications of MELS modeling to pain data. We will begin with a basic MELS model that only estimates variances and unique intercepts for individuals; this will give an opportunity to describe the MELS model, including how the inter- and intra-individual variance components are related to those in multilevel modeling. Next, we will discuss including random slopes, as we anticipate many researchers may need to detrend their data. Finally, we will discuss a MELS model with predictor(s) to account for differences in inter- and intra-individual variability.

**Supplementary Information:**

The online version contains supplementary material available at 10.1007/s10865-026-00638-6.

## Introduction

Intra-individual variability in many behavioral and medical constructs is of growing interest. Such constructs include pain (Bartley et al., 2018; Mun et al., 2019), sleep (Bei et al., 2016; Okun et al., 2011), cognitive performance (Mumme et al., 2021; Ram et al., 2005), and emotion (Gruber et al., 2013; Wang et al., 2021). Looking beyond mean differences and examining intra-individual variability provides an additional stream of information with theoretical and practical relevance. For example, examining intra-individual variability has been key in developing state-trait distinctions in various domains such as personality and emotion (Nesselroade and Ram, 2004).

While it makes both intuitive and theoretical sense to examine not only how processes operate on average, but also how they vary over time, determining how to model intra-individual variability is not so intuitive. Intra-individual variability is often quantified using the intra-individual standard deviation (iSD), or related measures like variance or the coefficient of variability (Berli et al., 2015; Farmer et al., 2022; Mezick et al., 2009). Although iSD captures some aspects of variability, it can be unstable and unreliable when calculated on a small number of observations and it necessarily collapses all within-individual observations (reducing statistical power) making it a statistically inefficient and unreliable approach in many limited data scenarios like ecological momentary assessment (EMA) and intensive intraindividual variability (IIV) studies (Estabrook et al., 2012). Multi-level models (MLMs; i.e., mixed effects models, hierarchical linear modeling) are a common approach for modeling data structures like those that arise from EMA studies and other similar intensive longitudinal designs. MLMs are sometimes talked about as if they capture within-individual variability, and while it is true that a major strength of MLMs is the ability to partition within and between individual variance^1^, MLMs do not explicitly model these variance components in relations to other variables.

One underused methodological tool for modeling variances that combines the benefits of iSD approaches and multi-level modeling is mixed-effects location scale (MELS) modeling (Hedeker et al., 2008), which is a multi-level approach that explicitly models variance components. MELS is preferable to variability metrics like the iSD because it is better powered and more reliable in scenarios with individual time series that are $<30$ observations in length (Walters et al., 2018). Unlike typical multi-level models, MELS allows for both fixed and random effects on both within- and between-individual variance components, which provides the ability to ask questions about inter-individual differences in intra-individual variability. Standard MLMs partition between and within variance components, but only allow predictors on the mean (location), whereas MELS allows for predictors on the mean and/or the variances (scale).

MELS achieves this by modeling residual variances in addition to modeling means, which allows researchers to more appropriately model data where within-individual variance differs across people (as this kind of data violates the homogeneity of variances assumption of standard MLMs). MELS is also an incredibly flexible modeling framework that offers the unique opportunity to model both within- and between-individual variance with either time-varying or time-invariant predictors, which can help us understand both whether, and potentially why, people vary more or less.

**Fig. 1:**
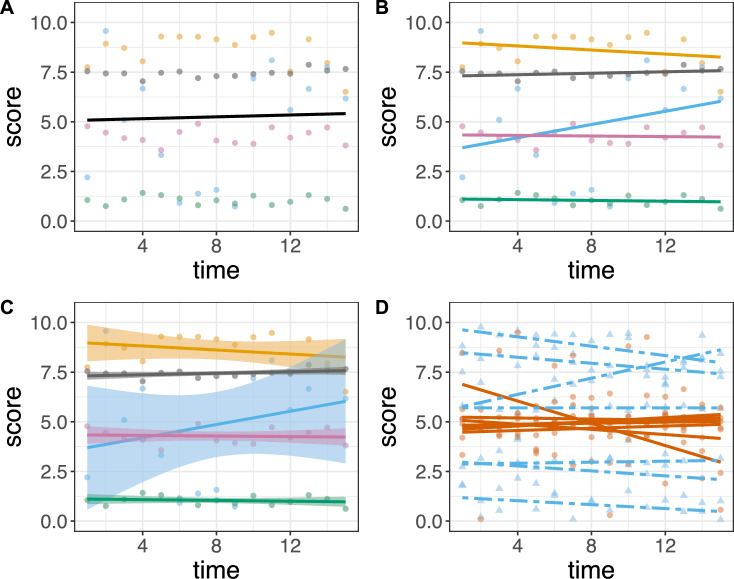
Visual depiction of differences in how A). ordinary least squares (OLS) regression, B). multi-level modeling (MLM), C/D). mixed-effects location scale (MELS) modeling would model data. In OLS regression (plot A) only one slope and one intercept (depicted by the black line) is modeled based on data points across all individuals. MLM (plot B) allows for a unique slope and intercept for each individual. MELS models also allow for a unique slope and intercept for each individual, but also different within- (plot C) and between- (plot D) individual variances. In plot C this is depicted by the shaded regions representing differing within-individual variances for different individuals. Plot D shows two different groupings of individuals (depicted by the blue/dashed and orange/solid lines) that have a differing amounts of between-individual variance. The orange/solid lines have lower between-individual variance and are more similar to one another, while the blue/dashed lines have higher between-individual variance and are less similar to one another. MELS can explicitly model these variance differences and put predictors on either the within- or between- individual variance components. For example, the blue/dashed and orange/solid lines could represent some dichotomous predictor that relates to the amount of between-individual variance

The present tutorial introduces the MELS model to health and psychology researchers with an application to a dataset on chronic pain symptoms over time. Across chronic pain conditions, higher pain variability is associated with negative outcomes including greater pain severity (Allen, 2007), depression (Bartley et al., 2018; Zakoscielna and Parmelee, 2013), lower perceived health (Zakoscielna and Parmelee, 2013), lower quality of life (Bakshi et al., 2022), ineffective coping responses (Wesolowicz et al., 2021), and greater responsiveness to placebo(Harris et al., 2005). Prominent theoretical models (e.g., the biopsychosocial model) also describe pain as highly variable and dynamic (Adams and Turk, 2018; Gatchel et al., 2007). Given this, there is growing consensus that variability in chronic pain symptoms needs to be more closely examined. However, similar to other areas of health-related research, most examinations of pain variability use the iSD or other questionable metrics like the mean-square of successive differences (Mun et al., 2019).While, the example in this paper is on ecological momentary assessment (EMA) data, MELS can be applied to any data with a nested structure (e.g., students within a classroom, trials within people). MELS is useful as it addresses many of the drawbacks of methods like the iSD, while retaining a similar logic. MELS is also a more flexible modeling framework that can readily handle data scenarios like differing numbers of observations per person. Thus, in scenarios like ecological momentary assessment (EMA) where shorter time series are common (due to the high burden of collecting more than 30–50 observations over time) MELS is a better statistical choice compared to the iSD Fig. 1.

This tutorial will walk through the MELS model and demonstrate software through a series of three increasingly sophisticated applications. We will begin with a basic MELS model that only estimates variances and unique intercepts for individuals; this will give an opportunity to describe the MELS model, including how inter- and intra-individual variance components are related to those in multilevel modeling. Next, we will discuss including random slopes, as we anticipate many researchers may need to detrend their data. Finally, we will discuss a MELS model with predictor(s) to account for differences in inter- and intra-individual variability. Supplementary materials including simulated sample data and more extensive descriptions of how to implement these models are available at https://osf.io/78jb3/.

## Data & software description

To demonstrate the utility of MELS modeling for health data we will apply various versions of the MELS model to a dataset on chronic pain (fibromyalgia) symptoms over time. This dataset contains 3X/day assessments of pain, fatigue, and emotional distress (each rated on a 0–6 Likert scale) for 7 days, alongside a battery of other one-time measures (e.g., anxiety diagnosis, opioid use) on 310 patients with fibromyalgia. Other measures and more methodological details can be found in Okifuji et al. (2010). While the original data cannot be shared as a part of this tutorial, similar simulated data is provided at https://osf.io/78jb3/, so that readers can practice implementing analyses similar to those presented here; detailed screen captures are provided to allow readers to follow the analyses step-by-step.

This tutorial will implement MELS models using MixWILD (Dzubur et al., 2020) (formerly MIXREGLS (Hedeker and Nordgren, 2013)), which is a standalone GUI-based freeware program, which can be downloaded at https://reach-lab.github.io/MixWildGUI/. MixWILD is sufficient for most MELS model implementations and doesn’t require programming expertise, making it more accessible for many researchers. In our supplementary materials https://osf.io/78jb3/, we provide a walkthrough of how to partially replicate the models presented in this tutorial within brms (Bürkner, 2017), for users who may prefer R. For more complex model specifications, MELS models can be manually programmed in R using Bayesian estimation via programs like JAGS and STAN (Rast et al., 2012; Williams et al., 2021) or specified via the brms package (Lester et al., 2021). These latter specifications are less user-friendly, and require more extensive programming expertise, but worth mentioning for users whose models may exceed the capabilities of MixWILD. That being said, the software options for MELS models are expanding with recent implementations capitalizing on the functionality built for DSEM within MPlus (Brose et al., 2024; McNeish, 2021) and an R package with latent variable modeling capabilities (Martin and Rast, 2022).

**Fig. 2:**
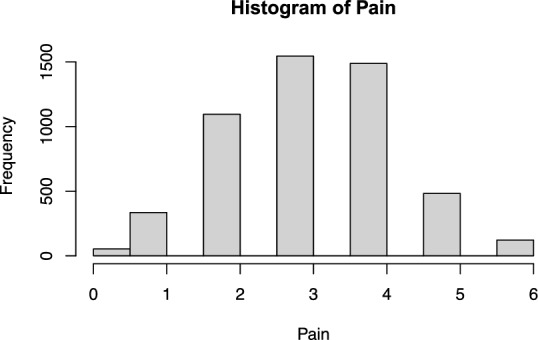
Histogram of the *Pain* outcome. While not addressing the categorical nature of the responses, the distribution has a skew of $-$ 0.05 and kurtosis of $-$ 0.21, which are much lower than values typically considered to indicate non-normality

It should be noted that before beginning the analyses, careful consideration was given to the characteristics of the primary dependent variable *Pain*. MixWILD allows for the dependent variable, later called the “Stage 1 Outcome”, to consist of Continuous, Dichotomous, or Ordinal outcomes. The pain data, in this case, are ordinal as they were measured on a Likert scale. As our scale has the minimum 6–7 categories which can produce similar results with both continuous and ordinal analyses (Rhemtulla et al., 2012), we examined the distribution of pain (See Fig. 2). Despite being measured on a discrete scale, the distribution of pain scores did not show hallmarks that would suggest the residual structure is far from normally distributed, as the shape of the distribution was symmetric and approximately normal. Consequently, for these data, modeling the data as continuous seemed reasonable, but careful consideration should be given before beginning to analyze one’s data.^2^

## Basic MELS

Examining intra-individual variability in health related constructs necessitates collecting repeated measurements within an individual. This creates a data structure where observations over time (e.g., pain at time 1, 2, 3,...) are nested within individuals and these observations within an individual are likely more similar than observations across individuals. For traditional regression approaches (e.g., linear ordinary least squares regression), this kind of nested data can violate the assumption of independent observations and be statistically inappropriate. Multi-level models (MLM, a.k.a., mixed models) can account for and explicitly model this statistical dependence, making them a more statistically sound choice for many nested data structures. There are many available resources on multi-level modeling (e.g., Raudenbush, 2002, Willett & Singer, 2003), and it is a statistical method with widespread applications in the behavioral and medical sciences.

MELS models extend the basic structure of MLMs to modeling variance components, and thus take a similar form. Using the example of differences in pain variance amongst fibromyalgia patients. The most basic MELS model, could be specified by the following equations, with i representing individuals and t representing observations:

1 $$\begin{aligned} Pain_{it}= & \beta _{0i} + \epsilon _{it} \end{aligned}$$

2 $$\begin{aligned} \beta _{0i}= & \gamma _{00} + {u_{0i}} \end{aligned}$$

3 $$\begin{aligned} \epsilon _{it}\sim & N(0, \sigma ^2_i) \end{aligned}$$

4 $$\begin{aligned} \sigma ^2_{i}= & exp(\omega _0 + u_{1i}) \end{aligned}$$

In which pain at some timepoint (*t*) in a given individual (*i*) is predicted by an intercept value ($\beta _{0i}$) that is allowed to differ for each individual as indicated by the *i* subscript. This intercept value in eq.  2 specifies both a fixed ($\gamma _{00}$) and random effect ($u_{0i}$). The fixed effect ($\gamma _{00}$) is the overall/grand mean of the sample, and an individual’s mean may deviate from that mean due to $u_{0i}$ (the estimated variance of $u_{0i}$ is $\tau _{00}$, expressed in Eq. 5). Eqs. 3 and  4 are where MELS models diverge from standard MLMs as the $\epsilon _{it}$ term is now explicitly modeled and has an *i* subscript on the variance component $\sigma ^2_i$. In a standard MLM there is no subscript on $\sigma ^2$ (i.e., $\epsilon _{it} \sim N(0, \sigma ^2)$), which means the residual variance is assumed to be homogeneous for all individuals and across all levels of any predictors in the model.

MELS models address this limitation of standard MLMs and provide an opportunity to explicitly model heterogeneous variances. The residual/error term $\epsilon _{it}$ which is modeled by eq. 3 depends on the within-individual variance $\sigma ^2_i$ term defined by eq. 4. Eq.  4 states that the variance of the residuals $\sigma _i^2$ is equal to the exponent of a fixed effect $\omega _0$ that is the same for all individuals, and a random effect $u_{1i}$ which allows the variance to be different for different individuals. As the variance $\sigma _i^2$ should not take negative values, MELS uses a log-linear model in which $\sigma ^2_i$ is modeled within an exponential function. This serves to keep the variances positive. In eqs. 1–4 the fixed (average) effects are $\gamma _{00}$ for the location and $\omega _0$ for the scale. The random (deviation) effects are $u_{0i}$ for the location and $u_{1i}$ for the scale. Eq. 5 expresses that $u_{0i}$ and $u_{1i}$ are assumed to be normally distributed with a mean of 0 and some variance ($\tau _{00}$ and $\tau _{11}$ respectively). The covariance ($\tau _{10}$) between $u_{0i}$ and $u_{1i}$ is also modeled within this same matrix.

5 $$\begin{aligned} { \begin{bmatrix} u_{0i}\\ u_{1i}\\ \end{bmatrix} \sim N\begin{pmatrix}\begin{bmatrix} 0\\ 0\\ \end{bmatrix}, \begin{bmatrix} \tau _{00} & & \\ \tau _{10} & \tau _{11} & \\ \end{bmatrix} \end{pmatrix}} \end{aligned}$$

**Fig. 3:**
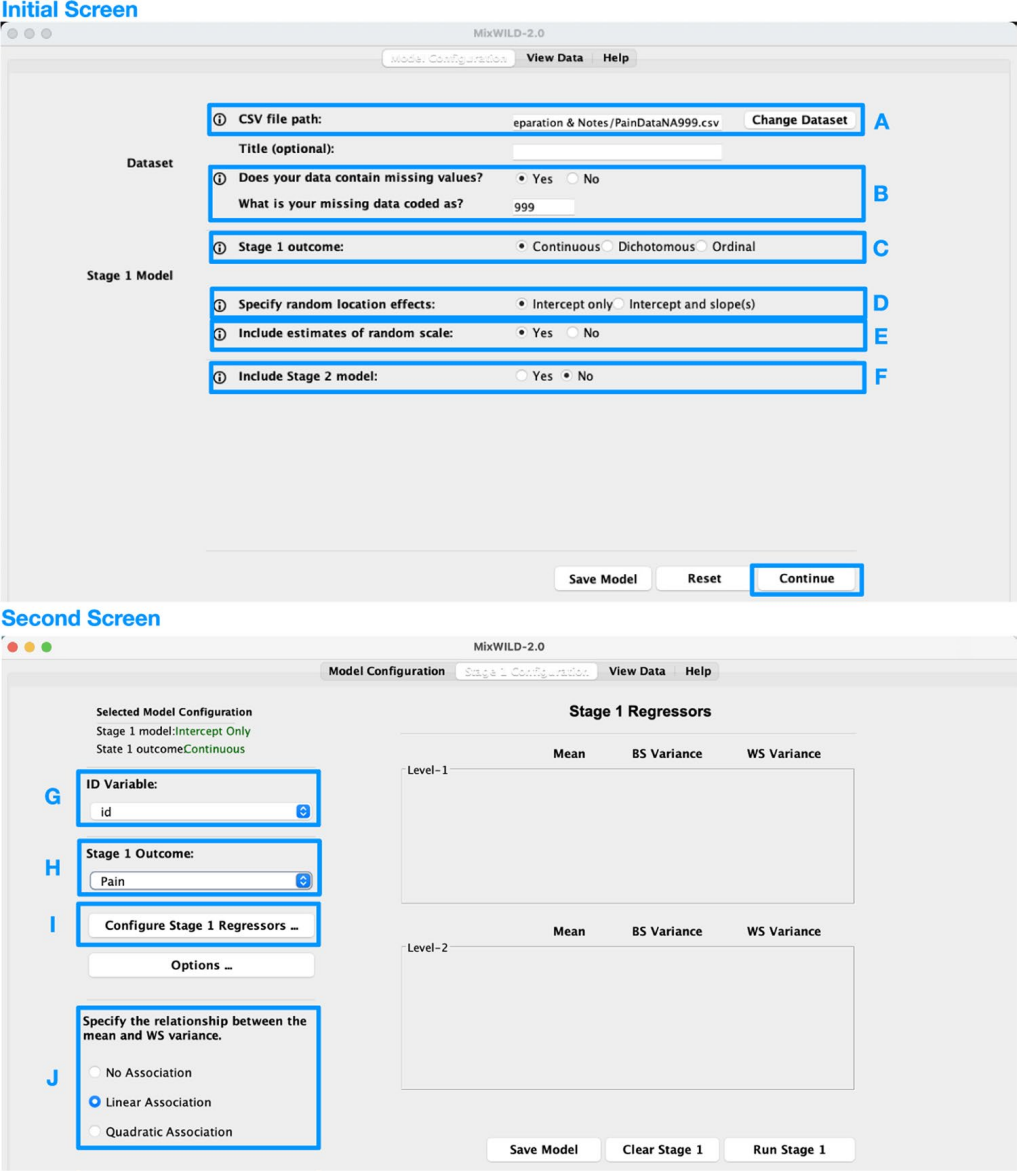
Sample screen captures of MixWILD v2.0. In the upper panel, the “Initial Screen” is presented after selecting one’s data. The lower panel is the screen the follows after pressing “Continue” on the initial screen. The highlighted boxes represent different inputs to the model, as discussed in the text

To create this model in MixWILD, the first step is to specify a CSV file path (Fig. 3A). As with most MLMs, the data should be in long format where repeated observations for the same individual occur on multiple rows, and an *id* variable indicates responses from differing individuals; the supplement includes a sample data file in long format. The next step is to specify if the data contains missing values, how missing data are coded (Fig. 3B), and if the outcome variable (in this case pain) is continuous, dichotomous, or ordinal (Fig. 3C). For this model, we will be treating our outcome as continuous, as discussed previously. Finally, for the stage 1 model (which is the only model needed to represent eq.1–3), specify random location effects (Fig. 3D; for this model only the intercept will be included, but in the following sections we detail how to run and interpret a model with random intercepts and slopes), include estimates of random scale (Fig. 3E, select "No" for the stage 2 model (Fig. 3F), and hit “Continue" (in the lower right hand area of the screen).This will advance to the “Second Screen" (bottom half of Fig. 3) to specify the ID variable (F3G), stage 1 outcome (Fig. 3H; for the current model “pain"), and the association between the mean and within-individual variance (Figure 3J). For this model, no predictors are being entered into the model, so nothing should be specified for Fig. 3 (later sections will detail how to enter predictors). This model should converge relatively quickly (<30 s), but for more advanced models, such as those detailed in the following sections, models can take several minutes to converge. It is worth noting that in MixWILD the option to model the between-individual variance component is only available in models that do not specify random effects for predictors (e.g., individual-specific slopes.) In this tutorial we will not be covering how to implement a stage 2 model (see Dzubur et al., 2020 for more details).

In the present model, we specify the relationship between the mean and within-subject variance to be a linear relationship, so as to not assume the covariance between these variances to be exactly zero. When working with iSD, it is not uncommonly expected that the mean and within-individual variance could be correlated; for example, as means increase, so does the variance, but at very low means, there may be floor effects that result in much lower variance. In this example, the variances would be linearly related and have a non-zero covariance. If we expected that both high and low means were associated with lower variance, perhaps due to both floor and ceiling effects, but that moderate means had higher variance, this covariance between means and within-subject variance could be modeled using the “quadratic association” option.

Before going through the results, we want to make a note about differing notations used to represent MELS models. Since many within the behavioral medicine community are familiar with multi-level modeling, we selected to introduce MELS using the notation in the prior equations, modeled after McNeish, 2021. However, MELS models were originally represented in a different equation format Hedeker et al. (2008). We reference both equation formats, but readers can use whatever format they prefer. In the Hedeker et al., 2008 format the model represented by Eqs. 1-5 would be represented as below with *i* representing individuals and *t* representing observations:

6 $$\begin{aligned} Pain_{it}= & \beta _{0} + \upsilon _i + \epsilon _{it}\end{aligned}$$

7 $$\begin{aligned} \upsilon _i\sim & N(0, \sigma ^2_\upsilon )\end{aligned}$$

8 $$\begin{aligned} \epsilon _{it}\sim & N(0, \sigma ^2_{\epsilon _i})\end{aligned}$$

9 $$\begin{aligned} \sigma ^2_{\upsilon _{}}= & exp(\alpha _0 )\end{aligned}$$

10 $$\begin{aligned} \sigma ^2_{\epsilon _{i}}= & exp(\tau _0 + \tau _\nu \nu _i + \omega _i) \end{aligned}$$

11 $$\begin{aligned} \omega _i\sim & N(0, \sigma ^2_\omega ) \end{aligned}$$

Where $\upsilon _i$ represents individual means for *i* individuals and $\sigma ^2_\upsilon$ represents the between individual variance. An (*i*) individual’s deviation from their personal mean at time (*t*) is represented by $\epsilon _{it}$, and $\sigma ^2_{\epsilon _i}$ represents the within-individual variance. Because MixWILD was build on this equation notation, it will be reflected in the output; for example, tau $\tau$ and alpha $\alpha$ will be used for within-individual (WS) and between-individual (BS) variance effects. So we include this alternative way to represent the equations as a reference.

After running the model in MixWILD, the output can be found on the “Stage 1 Results” tab. Scrolling down to the end of the MixWild output, we can see the following results:
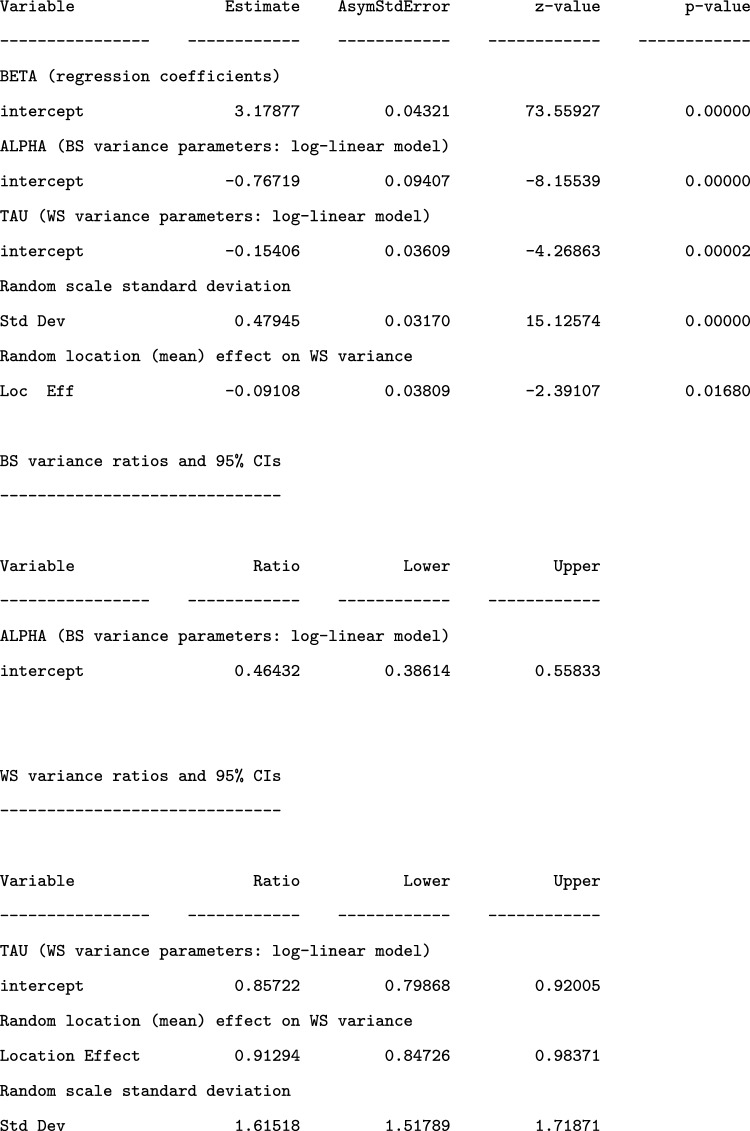


Starting with the “BETA (regression coefficients)" heading in the results (which is the fixed effect), the intercept here ($\gamma _{00}$ from Eq. 2 or $\beta _0$ from Eq. 6) is 3.18, which is the mean pain score across all observations and individuals. The significance test for intercept estimates in MELS (and multi-level models more generally) represents whether a given estimate differs from 0 (1 in the log-linear model). Thus, in substantive applications, the intercept values are unlikely to be of interest, but for the sake of the tutorial and gaining familiarity with the MELS model, we will walk through what each line on the output represents. For the between individual variance “ALPHA (BS variance parameters: log-linear model)", the intercept estimate is $-$ 0.77, which is the log of the estimated between individual variance (i.e., $\alpha _0$ from Eq. 9, or the log of $\tau _{00}$ from Eq. 5). Negative variances are meaningless and the only reason this value is negative is because of the log metric. Under the “BS variance ratios and 95% CIs" heading, there is an exponentiated version of estimates. Taking the exponent undoes the log function and transforms the estimates back into the original metric of the data. Thus, the estimate of the between individual variance in pain ratings among the 310 fibromyalgia patients in this sample is 0.46 (i.e., $exp(-\,0.77)$). For the within individual variance, “TAU (WS variance parameters: log-linear model)" the intercept estimate is $-$ 0.15. This is represented as $\tau _0$ in Eq. 10 and $\omega _0$ in Eq. 4. The exponentiated within individual pain rating variance estimate is 0.86 (i.e., $exp(-\,0.15)$), which can be found under the "WS variance ratios and 95% CIs" heading of the results output.

The output lines “Random scale standard deviation" and “Random location (mean) effect on WS variance" are of particular substantive interest. The “Random scale standard deviation" output summarizes a statistical test of if individuals have significantly different within-individual variances in their pain ratings (standard deviation of the within-individual variance random-effects, $u_{1i}$) in the log metric. For this sample, within-individual pain rating variance differed significantly (*z*=15.13, $\textit{p}<.001$), implying fibromyalgia patients pain ratings vary in their consistency/erraticism over time. Finally the “Random location (mean) effect on WS variance" shows a significant negative effect (*z*=$-$ 2.39, $\textit{p}=.02$) of $-$ 0.09, implying that fibromyalgia patients with higher average pain ratings show more consistency in their pain ratings over time. Even the simplest version of this model adds additional functionality beyond a typical multi-level model in that we are now able to test whether there are individual differences in variance.

## Controlling for random slopes

The prior section introduced the basic MELS model. The model included a random location effect (i.e., random effect in MLM), to allow for differences in individual mean pain scores. The model also included a random scale effect, which is unique to the MELS model; this effect allows the within-individual variance to differ thus allowing individuals to differ in the variance of their pain scores around their individual mean. This basic model most closely resembles iSD estimates, as for each individual the variance of scores around an individual mean is estimated.

When iSDs are calculated on longitudinal data, or data on many trials (e.g., Hultsch, MacDonald, Hunter, Levy-Bencheton, & Strauss, 2000), it may be preferable to remove data trends associated with time or trial number. If over the course of one or more weeks of pain data there may be some progression leading to an increase or decrease in pain over the study period, it may be desirable to remove this trend. Similar considerations may be true for measures that could show learning or practice effects. If one considers more progressive changes over a study period as representing intraindividual growth or development, removing of these trends is sometimes necessary to partition out the variance that might be best characterized by intraindividual variability (Nesselroade et al., 1991). The present section modifies the basic MELS model to account for differences in individual growth trajectories.

To model changes in time, one common approach is to incorporate a predictor that serves as an index of time; while modeling a linear effect of time is more common, other more complicated effects like higher order polynomials can be considered (e.g., quadratic effect which is related to acceleration; Deboeck, Nicholson, Kouros, Little, & Garber 2015). As with MLMs of longitudinal data, it is commonplace to model data allowing individuals to have unique intercepts and unique effects of time through the inclusion of random effects. For a linear effect of time, the MLM would be

12 $$\begin{aligned} Y_{it} = \beta _{0i} + \beta _{1i} (Time) +\epsilon _{it} \end{aligned}$$

with *i* and *t* indexing different individuals and observations respectively, and the *i* subscripts on the regression coefficients indicating that the intercepts $\beta _{0i}$ and slopes related to time $\beta _{1i}$ differ for different individuals. In MLM, a set of level 2 equations is usually included, such as $\beta _{1i}=\gamma _{10} + u_{1i}$, to highlight that there is a fixed effect $\gamma _{10}$ that is estimated, but that individuals may uniquely differ from the fixed effect due to the inclusion of a random effect $u_{1i}$.

Because MELS models are built on the MLM framework, the inclusion of random effects associated with predictors (e.g., time) can be readily incorporated. Compared to the basic model in the prior section, the primary changes include first indicating that there will be random slopes in addition to random intercepts; these are specified as part of the location effects (Fig. 3D). Subsequently, (Fig. 3, “Second Screen”), one configures the stage 1 regressors (Fig. 3I). This will allow the addition of time as a level 1 (Time varying) predictor, as the values of time will vary within each individual for the repeated observations on days 1 through 7 in the pain data. Within the Level-1 box several check-box options will appear; these include “Mean,” “Random Slope,” and “WS Variance.” Checking “Mean” for the time predictor will include the fixed effect of time $\gamma _{10}$; this fixed effect allows the effect of time for the entire sample of individuals to have a mean that differs from zero. Checking only “Mean” would assume that the slope associated with time was the same for all individuals (i.e., parallel trajectories), so to allow individual slopes due to time to vary the checkbox for “Random Slope” for the the time predictor must also be selected; this adds the random effect for time $u_{1i}$.

After running the model, scrolling to the end of the “Stage 1 Results," the pain data results include:
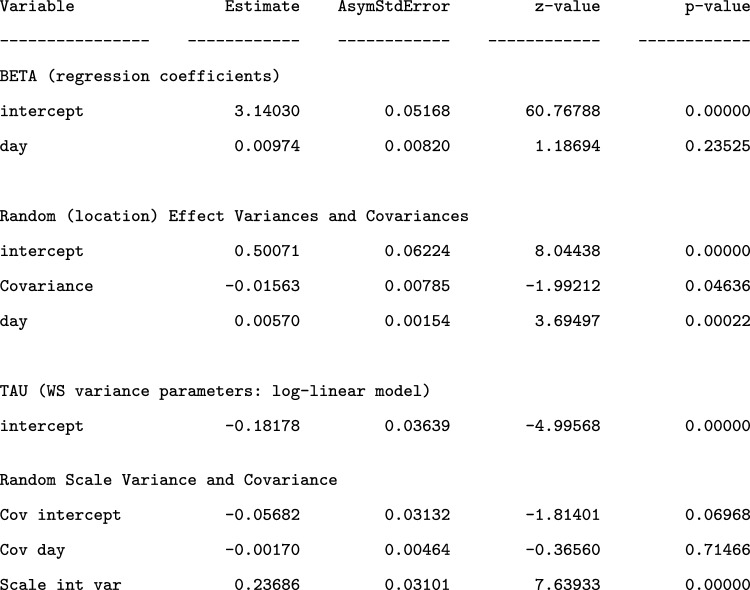


In this table the interpretation of the *day* effects, our index for time, are the same as with a MLM. Under “BETA (regression coefficients)” are the fixed effects. The present data highlight that in this sample of individuals, on average the change in pain over seven days does not significantly differ from zero (*p* =.24); the estimate for change in pain for each additional day in the study is very small (0.01) relative to the scale (which varies from 0 to 6). The effect listed for *day* under “Random (location) Effect Variances and Covariances)” estimates the variance of the slopes to be 0.006; taking the square-root of this number provides a standard deviation for the slopes of 0.075. Taken together, this highlights that while on average there is not a change in pain related to days, there may be some individuals showing change. Individuals at 1 standard deviation above the mean would have a slope of 0.08524 ($0.00974+1\sqrt{0.00570}$); over 7 days this slope would result in a change of 0.597 ($0.08524*7$), which could be notable given the range of the scale.

Comparing the results with the prior basic model, one will see changes in the within-subject variance parameters from $-$ 0.154 to - .182, corresponding to variances of 0.86 (i.e., $exp(-\,.154)$) and 0.83 (i.e., $exp(-\,0.182)$) respectively. While a small change, part of the within-subject variance has been extracted and attributed to change across days. Consequently, the decision whether to control for random slopes is an important substantive consideration as inclusion or not of these effects changes what is attributed to “intraindividual variability.” In the pain data considered here, changes related to time across the one-week of data are more likely related to variation due to weekly patterns rather than a long-term change in pain; as such, the variance due to differences in linear slopes seems more likely to be an important component of intraindividual variability that it would not be desirable to extract from these data. But the example serves to demonstrate that the detrending sometimes applied to iSDs can also be accomplished within the MELS framework. In addition to detrending, there may be other time varying or time invariant variables (e.g., group differences) that contribute additional variance, which it may be desirable to extract from the data. The pain data, for example, include multiple measures within the same day; if there was reason to expect that there might be additional, systematic variance due to morning/noon/evening measurement, that variance could be modeled and extracted from the within-subject variance. It is an essential substantive question, however, whether all within-individual variance should be counted as intra-inidividual variability, or whether the variance of slopes across time, group differences, time-of-day effects, or other variance should be excluded from the estimates of an individual’s variability.

Also not considered in this example, but essential with any multilevel model, is how the data are centered and the subsequent impact on interpretation. In the current example, one could consider centering *day* differently, including grand-mean centering. In the present data, day is coded from 1 to 7. The estimate of the location intercept “BETA”, the random location effect intercept, and the random location effect covariance all correspond to values when *day* is zero, which is outside the range of the data. Rescaling *day* so that the first day is zero, the middle day is zero, or grand-mean centering on days with observed values would each have a different effect on the interpretation of the lower-order terms in the model. If the model were to add predictors of intercept differences, or of between-subject differences in variance, the selection of how day is coded would have implications for interpretation. While all possibilities are not described in detail here, we wanted to highlight the different centering approaches that could be employed in this framework.

## Modeling within subject variance

The prior models are perhaps most similar to common applications of iSD. The basic MELS model estimates individual variances around individual means, and the second example considers the estimation of the within-subject variances partitioning out variance due to differences in individual slopes or other predictors. While individual estimates of variance could be extracted and subsequently modeled, such two-step procedures tend to be statistically inefficient. In this final example the full utility of MELS modeling for characterizing intraindividual variance is considered. In this section, the MELS model is used to examine whether intraindividual variance systematically varies with time-invariant differences between individuals as well as time-varying covariates within an individual.

The modeling of time-invariant predictors parallels the idea of regressing iSD onto between-individual differences; in the pain data these could be hypotheses that individuals that use opioids or are not diagnosed with an anxiety disorder may demonstrate lower variance in their perceived pain ratings. Such models of between-individual, time-invariant predictors of variance differences modify Eq. 4 to include predictors of the within-individual variance $\sigma ^2_{i}$, such that:

13 $$\begin{aligned} \sigma ^2_{i} = exp(\omega _0 + \omega _1(OpioidUse_i) + \omega _2(Anxiety_i) + u_{{1i}}) \end{aligned}$$

where $OpioidUse_i$ and $Anxiety_i$ are used as examples of covariates measured once for each individual to address whether using opioids (a 0/1 predictor) or having an anxiety disorder diagnosis (0/1) is associated with differences in individual variances $\sigma ^2_{i}$; naturally, continuous predictors can also be considered.

The latter possibility of including time-varying covariates provides the opportunity to address questions that are not readily addressed with iSD. Rather than between-individual differences, MELS models allow researchers to address whether there are differences in variances related to a covariate which varies within an individual. For example, one could ask whether intraindividual variance in pain changes across the study duration, perhaps due to the application of an intervention, through the inclusion of *day* as a predictor of within-subject variance. In such a case, the within-individual $\sigma ^2_{i}$ variance would vary by both individual and time, that is $\sigma ^2_{it}$; Eq. 3 would be altered to allow for these changes in variance across time, $\epsilon _{it} \sim N(0, \sigma ^2_{it})$. More importantly, however, is that the time varying *day* predictor could be incorporated into a variation of Eq. 4,

14 $$\begin{aligned} \sigma ^2_{it} = exp(\omega _0 + \omega _1(day_{it}) + u_{1i}). \end{aligned}$$

Like Eq. 4, there is a fixed-effect intercept when all covariates are equal to zero $\omega _0$. The subsequent fixed effect $\omega _1$ would address whether there is a systematic change in variance related to the time-varying predictor representing the day of the study. As before, the random effect $u_{1i}$ allows individuals to have differing amounts of variance not accounted for by the fixed effects and predictor(s). In the pain data being examined, one could question whether fatigue, which varies within individuals every day, is related to the variance of individual pain scores by using fatigue as a predictor rather than *day*. When calculating iSDs, the available observations are usually too limited to make multiple estimates representing differing levels of fatigue or the progression across a study, which is why including time-varying covaraiate provides an opportunity for addressing unique questions with MELS models.

### Time-invariant, dichotomous predictors

We begin by adding Opioid use and Anxiety diagnosis, both time-invariant, between-individual differences, to the basic MELs model. While the example in this sect. (5.1) uses dichotomous predictors available in the data, continuous time-invariant predictors of between-subject differences could also be considered; the interpretation of a continuous predictor is considered in the example in the next sect. (5.2). Given there was no substantial trend in the current data, the models presented in this section do not include the fixed or random effects of the previous section (i.e., day is not included as a predictor). As with the random slopes, we “Configure Stage 1 Regressions" (Fig. 3I) and add *Opioid* and *Anxiety* as level-2, time-invariant predictors. This presents the user with the option to include Opioids and Anxiety as predictors of “Mean”, “BS Variance”, and “WS Variance”; we include the predictors for all three, and will differentiate what each predicts in the context of the results.

After running the model, scrolling to the end of the “Stage 1 Results," the pain data results include:
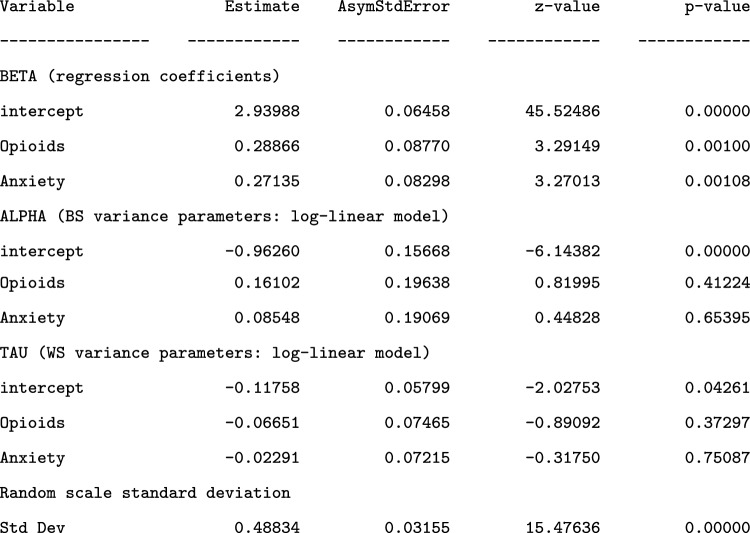


The “BETA (regression coefficients)” estimates represent the effects of Opioids and Anxiety when entered into a level-2 equation, as in Eq. 2. These give insight as to whether individual differences in intercepts are systematically related to predictors. In this sample, having been prescribed Opioids is associated with higher pain scores ($\beta =0.29$, $\textit{p}=.001$); similarly, having a diagnosis of an anxiety disorder is also associated with higher pain scores ($\beta =0.27$, $\textit{p}=.001$). The “ALPHA (BS variance parameters)” results allow one to consider whether the variance associated with the random intercept $u_{0i}$ differs based on a between group difference; these estimates would be useful if there was a hypothesis that the means of pain for different individuals showed higher variance in the group with Anxiety than the group without Anxiety, for example. In the present data, the means of individuals do not show more variance based on whether they have been prescribed opioids ($\textit{p}=.41$) or whether they have been diagnosed with an anxiety disorder ($p=.65$).

It is the section, “TAU (WS variance parameters)”, which finally addresses the question of interest common to applications of iSD: are individuals not taking opioids or with an anxiety diagnosis showing more variance? In the present data individuals taking opioids or not do not differ in their variance across the 7 days examined ($\textit{p}=.37$); individuals with and without anxiety disorders also do not show a difference in within-individual variance ($\textit{p}=.75$). While not significant, it is important to note that both the “ALPHA” and “TAU” entries are listed as “log-linear” models. Consequently, the interpretation of parameters has parallels to the interpretation of logistic regression parameters. The non-significant effect of opioids ($\omega _1=-\,.067$) would be easiest to understand after taking the exponent (i.e., $exp(-\,.067)$), resulting in a value of 0.935. This conveys that when changing *Opioid* from a value of 0 to a value of 1, the variance $\sigma ^2_i$ is *multiplied* by 0.935. If this were significant, this would suggest that the group receiving opioids ($Opioid=1$) shows 6.5% less within-subject variance than the group not receiving opioids. That is, like logistic regression, the parameters under “ALPHA” and “TAU” can be interpreted as the multiplicative effect on the variance estimates for a 1-unit change in the predictor, once the exponent of the estimate is calculated. MixWILD provides these exponentiated values, and their confidence intervals after the table discussed above under sections labeled “BS variance ratios and 95% CIs” and “WS variance ratios and 95% CIs.” For the present example, the table for WS variance was:
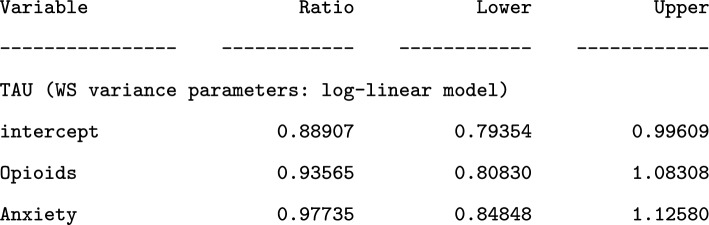


where the Opioids “0.93565” ratio is the value calculated (i.e., $exp(-\,.067)$), with a 95% confidence interval of 0.81 to 1.08; the inclusion of “1” in this confidence interval corresponds to a non-significant result, as if the ratio of the Opioid group variance and Non-Opioid group variance is 1, the variances would be the same.

As with the prior example, one should consider the centering of the predictors. In this example, as the predictors are dichotomous, using codes of zero and one let themselves readily to direct interpretation of the effect. However, many researchers with the same data may consider another coding, such as effects coding. Naturally, if the predictor were continuous, centering becomes essential to consider because of how interpretation of lower-order effects will be affected. In the subsequent example, where a time-varying, continuous predictor is used, we will person-mean center to disentangle the effect between and within subjects.

### Time-varying, continuous predictor

Now, we add a time-varying covariate, *Fatigue*, as time-varying, continuous (Likert, 0–6), predictor of within-individual variance to the basic MELS model; the present model will not include random slopes or the prior time-invariant predictors. Using "Configure Stage 1 Regressors", *Fatigue* is added as a Level-1 time-varying predictor. This presents the user with the option to include *Fatigue* as a predictor of “Mean”, “BS Variance”, and “WS Variance”. There is also the critical decision whether *Fatigue* should be disaggregated into within-subject and between-subject effects (Curran and Bauer, 2011); this person-mean disaggregation of time-varying predictors into within- and between-individual effects is a common choice to separate effects which can otherwise be confounded. As before, we will include the prediction for all possibilities (Mean, BS Variance, WS Variance), including disaggregation for all three, so that the questions that are addressed can be differentiated in the context of the results.

After running the model, scrolling to the end of the results file, the pain data results include:
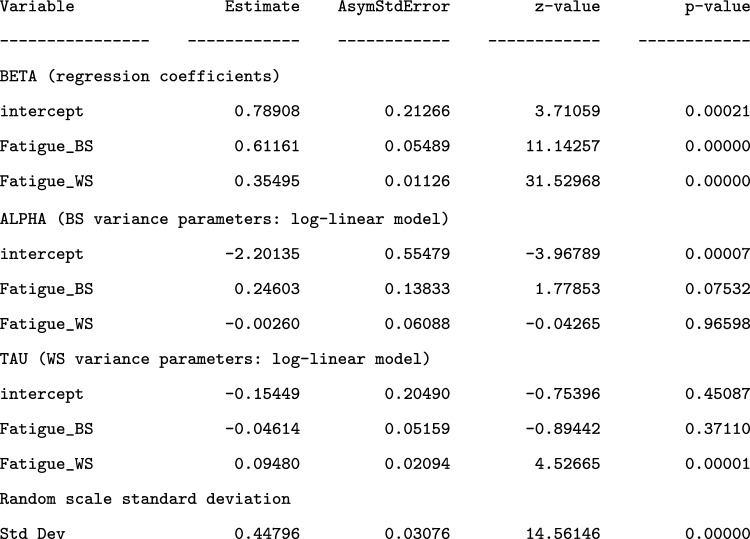


As when interpreting a MLM, under “BETA” the “Fatigue_BS” ($\beta =.61, p<.001$) conveys that people who are are more fatigued on average (i.e., imagine the mean of *Fatigue* for each individual) tend to have higher ratings of pain; “Fatigue_WS” ($\beta =.35, \textit{p}<.001$) conveys that whether an individual is above or below their personal *Fatigue* mean tends to be positively correlated with pain scores. One offers an interpretation of whether pain scores are correlated with between-individual differences in the individual averages for the predictor, while the second considers whether being above or below one’s personal mean is correlated with pain. As with the Opiod and Anxiety example, the “ALPHA” results convey whether the random intercepts (i.e., between-individual intercepts, $u_{0i}$ in Eq. 2) systematically vary with *Fatigue*. “Fatigue_BS” ($\alpha =0.246$, $\textit{p}=.075$), while not significant, produces a parameter that suggests with a larger sample we may see that individuals that have higher means for *Fatigue* may differ more in their intercepts, as each unit increase in mean *Fatigue* multiplies the random intercept variance by 1.28 (i.e., exp(0.246)). The inclusion of “Fatigue_WS” under “ALPHA” suggests an unusual question where the variance of random intercepts changes depending whether one is above or below one’s personal *Fatigue* mean.

The “TAU” section is where we examine whether the individual differences in variance are related to *Fatigue*. “Fatigue_BS” examines whether mean differences in *Fatigue*, that is those reporting more or less fatigue on average, are correlated with differences in intraindividual variance; in these data, it does not appear that mean, between-individual differences in *Fatigue* are related to intraindividual differences in variance ($\omega =-\,0.046$, $\textit{p}=0.37$). The “Fatigue_WS” examines whether being more or less fatigued at a particular moment is associated with increased variance in pain within individuals; this was statistically significant ($\omega =0.095$, $\textit{p}<.001$), with increased fatigue associated with increases in pain variance. This result reinforces that fatigue is an important and underexamined symptom within fibromyalgia (Vincent et al., 2013) Taking all of the results together: 1) being more fatigued on average is associated with higher pain scores (BETA, Fatigue_BS), 2) being more fatigued than one’s personal average at a particular moment is associated with higher pain scores (BETA, Fatigue_WS), and 3) and being more fatigued than one’s personal average at a particular moment is associated with increased variance in pain scores. Using MELS offers an unprecedented opportunity to disentangle not only how time-varying variables increase or decrease outcomes, but also offers insights as to how between- and within-individual variance differs in relation to predictors.

## Conclusions

The present tutorial introduced the functionality of the MELS model through three increasingly sophisticated applications. The first section (Basic MELS) introduced how MELS models extend the logic of the iSD into a more flexible and efficient modeling framework. As well as discussing how MELS models relate to typical MLMs, and the additional components included in MELS models. The second section (Controlling for Random Slopes) explored how random slopes function within MELS and demonstrated how they can be used to extract time-varying effects (e.g., detrend/remove trends associated with time). The third section (Modeling Within Subject Variance) demonstrated the full utility of MELS by illustrating how both time-varying and time-invariant predictors of both within- or between- individual variance can be incorporated. For the sake of length and readability, the details of MixWILD were kept relatively brief in an effort to prioritize conceptual understanding and building familiarity with how to interpret the results of various MELS models. However, more implementation details, extensive screenshots, and example data are available at https://osf.io/78jb3/.

Many of the topics/constructs studied within behavioral medicine (e.g., sleep, pain, stress, emotional state, smoking behavior) vary over time. Modeling of variances offers the opportunity to ask novel research questions within behavioral medicine that cannot be addressed with tests of mean differences. However, commonly used intra-individual metrics (like the iSD) have substantial limitations for many of the common data structures (e.g., EMA data with $<\,30$ observations per individual) in behavioral medicine. This tutorial provides a walk-through of MELS modeling, which is an alternative approach to modeling intra-individual variability that addressees the power and reliability limitations of metrics like the iSD (Estabrook et al., 2012).

There are some important considerations and limitations to keep in mind when using MELS models. MELS models can accommodate relatively small sample sizes (Hedeker et al., 2008; Williams et al., 2019), and are far preferable to the iSD in limited data scenarios. However, the combinations of small sample size and short time series (e.g., 20 individuals with 5 observations each) can lead to convergence issues (Hedeker et al., 2008), and statistical power remains an issue researchers should consider when implementing MELS models (Walters et al., 2018). It is also important to note that MELS models (as implemented in this article) do not differentiate between variability due to true variation in an underlying construct and measurement error (Rast and Ferrer, 2018), though there is some recent work that has begun to expand MELS into the latent variable space (Blozis, 2022; Nestler and Blozis, 2024). Further, appropriate handling of missing data within MELS models is still in development (Lin et al., 2018). Finally, MELS modeling is not sensitive to the ordering of observations within a time series (Deboeck et al., 2009), and thus does not capture the patterning of observations over time. Two individuals could have the same within-individual variance, but very different patterning over time and MELS modeling would treat these two individuals as if they were identical. While MELS modeling can accommodate the inclusion of time point as a predictor, which can control for a general trend in the data, it does not capture how observations relate to one another (e.g., auto-correlation). For research questions where this patterning may be important, other more temporally sensitive approaches (e.g., auto-correlation/regression, derivative estimation, difference/differential equation modeling) may be more appropriate.

While it is important to keep these considerations and limitations in mind, MELS models open up a whole host of new research questions. MELS models allow researchers to examine variability both within- and between- individuals, and explicitly model this variability by including variables that may account for either kind of variability (between- or within-individual). With this capability researchers can begin to understand not only how individuals vary, but why they vary. Within the context of the pain dataset examined in this tutorial, our analyses showed that fatigue is an important variable to consider when looking at pain variability. In FM patients, being more fatigued than average was associated with higher pain and *more variable* pain. This highlights that fatigue may be an important factor that contributes to higher and more variable pain experiences, and could be an important target in the treatment of fibromyalgia. The questions about variance that can be addressed with MELS models could transform our understanding of important psychological and health processes.

## Supplementary Information

Below is the link to the electronic supplementary material.

Supplementary file 1 (pdf 6023 KB)

## Data Availability

Data are available upon request. Materials and simulated data are available at https://osf.io/78jb3/

## Footnotes

1 Intra/within and inter/between are often used synonymously as are individual/subjects when describing MELS models.

2 It should be noted that in order to ensure the statistical conclusion validity of the inferences and to err on the side of caution the authors re-ran the models of within-subjects variance (sect. 5) with the outcome as ordinal, and compared these to the results from models with the outcome as continuous. This helped ensure that the inferences made were not dependent on the assumption that pain could be modeled as continuous. While the estimates differed because of difference in the underlying models, a comparison of statistical significance for the effects of Anxiety and Opioids in the first within-subjects model indicated that both the ordinal and continuous analyses result in the same inferences for these predictors. Both models show highly significant effects in the location model (*p* .001 both predictors, both models), and similar non-significant *p*-values for the effects of Anxiety or Opioids on between-subject or within-subject variance. It was not possible, to fully replicate the analysis of the model with fatigue. Convergence issues occurred when using the ordinal outcome in MixWILD with fatigue disaggregated into between-subject and within-subject components. In our estimation, for these data and the fatigue model, it seems a lesser misspecification to assume a continuous outcome than to present a model where the time-varying predictor is not disaggregated because of the known problems with multilevel model interpretation when one does not separate between-subject and within-subject components. Continuous-outcome models are presented, as we anticipate that these analyses will be more common and allow for a more straightforward interpretation.

## Code availability

https://osf.io/78jb3/
