# Supplementary material for: How am I doing? It varies: mixed-effects location scale modeling to examine intra-individual variability in health-related research
Source: J Behav Med. 2026 Feb 20;49(2):239–53. doi: 10.1007/s10865-026-00638-6 (PMC13253709; doi:10.1007/s10865-026-00638-6)

SUPPLEMENT: User Example

The primary paper uses an example based on pain data that cannot be shared for the present tutorial. Acknowledging the value of a working example when learning new methods, this supplement steps through the same analyses presented in the paper using a simulated data set that can be shared. The simulated data are based on characteristics of the pain data presented in the paper, including the same variable names, but were generated using a simulation and in no way represent real individuals or real pain processes.

The file "JBMED_MELSTutorial_Supplement_FAKEData.csv" contains data consisting of 250 simulated individuals with 21 observations per simulated individual across 7 days. Data were generated to have missing completely at random observations at rates similar to the original data; the missing data are denoted with "999." The data are structured in the long data format, where each individual will have multiple rows of data corresponding to the repeated measurements across time. The variables consist of:

- id: A 1-250 index identifying each simulated individual.
- day: A 1-7 index corresponding to the 7 days of measurement.
- Pain: A 0-6 variable representing pain ratings for each simulated individual. Given the skew, kurtosis, distribution, and number of categories, it will be treated as continuous. This is the primary outcome.
- Tired: A time-varying covariate representing the fatigue of the simulated individual.
- Anxiety: A dichotomous, time-invariant covariate.
- Opioid: A dichotomous, time-invariant covariate.

The document is divided into the following sections:

- 1.0 MixWILD
  - 1.1 Basic MELS
  - 1.2 Model with Random Slopes
  - 1.3 Modeling Within Subject Variance
- 2.0 brms package in R
  - 2.1 Basic MELS
  - 2.2 Model with Random Slopes
  - 2.3 Modeling Within Subject Variance
- 3.0 Checking Convergence

The sections within each program correspond to the sections of the paper.

1 MixWILD

MixWILD is a stand-alone program available from `https://reach-lab.github.io/MixWildGUI/`. The subsequent screen images and results were generated using MixWILD version 2.0.

1.1 Basic MELS

MixWILD is available from `https://reach-lab.github.io/MixWildGUI/`. The subsequent screen images and results were generated using MixWILD version 2.0. The following screens capture the settings that were used in the primary paper, and to generate the results for the supplement. Users interested in replicating the analyses for the paper can follow the images that follow, replicating how they appear on their own computers, to generate the supplement results provided.

On opening MixWILD, users will select their data using "Start with New CSV File."

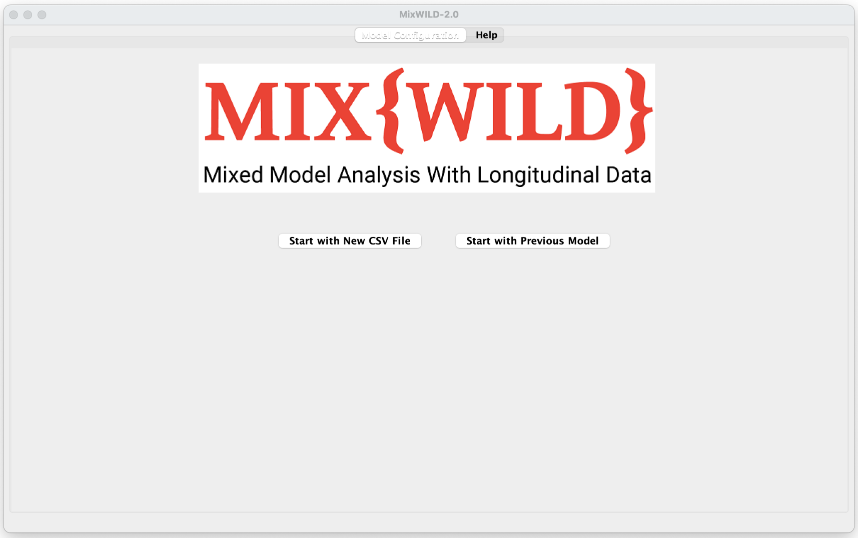

This will produce a pop-up window, from which one's CSV file can be selected.

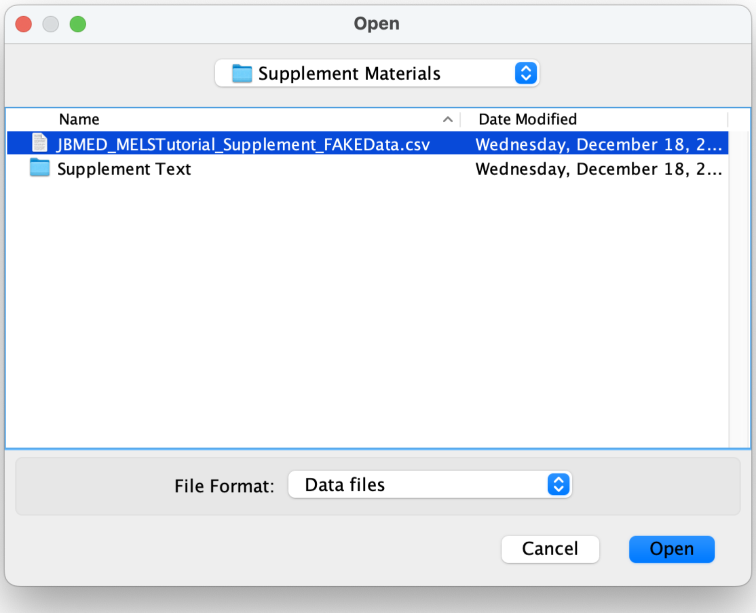

The first screen for specifying the model will then appear (pictured below). After providing a title (optional), one can provide information about whether there are missing data and how the missing data are coded. It is in the "Stage 1 Model" that several key choices are made. The first, "Stage 1 outcome," informs MixWILD whether the outcome is "Continuous," "Dichotomous," or "Ordinal." Data that are appropriate for multilevel data will often be treated as continuous, although with a large sample size data such as Likert scale data may be treated as ordinal. The next decision is to "Specify random location effects" which informs MixWild whether there will be random effects, like in Multilevel Modeling, for just the intercept or whether there will also be random effects for slopes; most introductory texts on Multilevel Modeling, Mixed Models, or Hierarchical Linear Models will address random effects, including random effects for intercepts and slopes. The final decision is whether to "Include estimates of random scale." It is the inclusion of estimates of random scale that allows the residual variance within individuals to differ; users interested in iSD will often be interested in selecting "Yes" to this option, as otherwise residual variance for individuals will only vary as a function of within-subject variance predictors and not allow to completely vary for different individuals. For data sets with a very limited number of observations per person, however, it may not be possible to estimate unique variances for each individual. The inclusion of Stage 2 models is beyond the scope of the

presented paper, but allow for extensions of the models presented here; for the models in this paper, "Include Stage 2 model" can be set to "No."

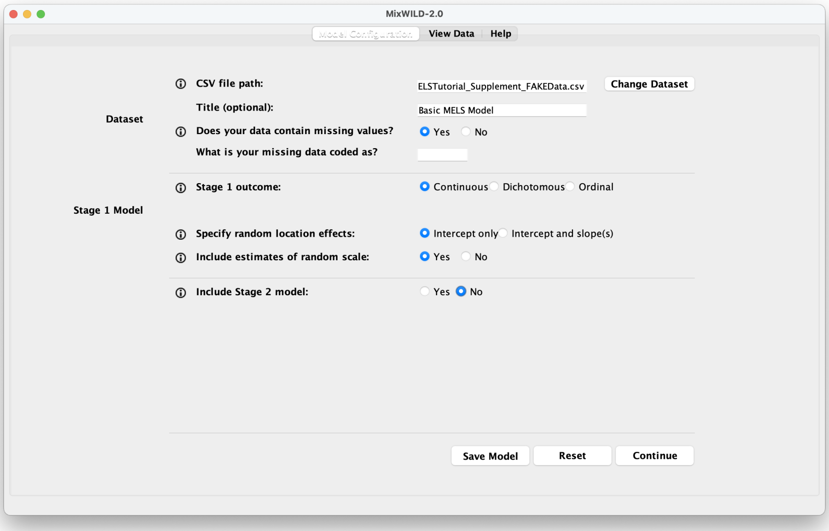

Once all options are selected, the "Continue" button will appear in the lower right corner; pressing "Continue" will bring up the second screen (pictured below). It is on this screen that the variables are identified. "ID Variable" and "Stage 1 Outcome" provide pull-down menus to identify the cluster/grouping variable and the outcome being models, respectively. Selecting "Configure Stage 1 Regressors ..." allows for the selection of time-varying and time-invariant predictors, which are discussed further in subsequent sections but not necessary for the Basic MELS model.

The primary choice on this screen, beyond identifying the ID and outcome variables, is whether one should "Specify the relationship between the mean and WS variance." When working with iSD, it is not uncommonly expected that the mean and within-person could be correlated; for example, as means increase, so does the variance, but at very low means, there may be floor effects that result in much lower variance. It is substantive considerations like this that could be seen as contributing to the development of the coefficient of variation, where the standard deviation of scores is divided by the mean, under the expectation that they will be related. Similarly, the MELS model allows the means and within-person variance to be uncorrelated ("No Association"), linearly related as in the prior example ("Linear Association"), or to have a "Quadratic Association." The last option could be considered for the pain example presented in the paper, as floor and ceiling effects in pain measurement may cause individuals with both very low and high pain to show the least variance. Whether there is a reason to expect a correlation between means and within-person variance estimates is both a substantive considera-

tion but also requires consideration of the measurement scale as it is applied to one's population of interest.

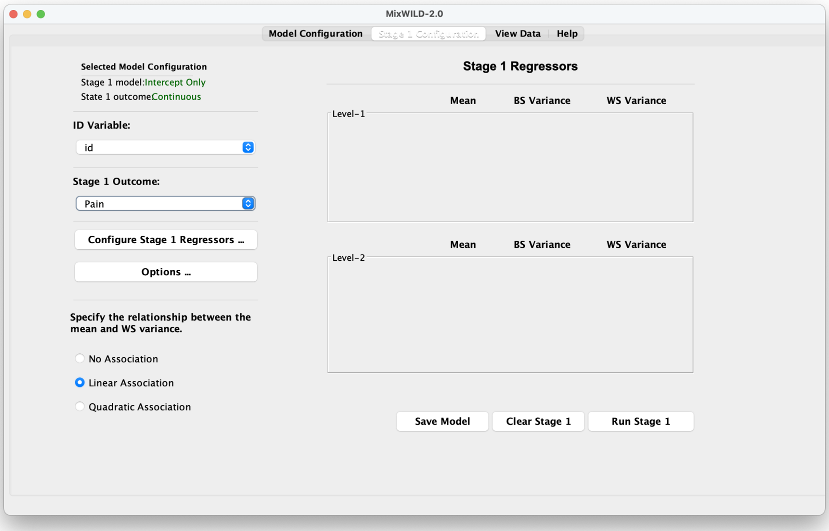

Following the selection of options in the prior two screens and pressing "Run Stage 1," a "Definition File Preview" screen (left, below) will appear, which allows one to save the definition file before selecting to "Proceed" with the analysis. Once one proceeds, one will be asked to "Please wait..." which calculations zoom by (right, below). The calculation stage can take a non-trivial amount of time, and walking away to get coffee is advisable for more complicated models.

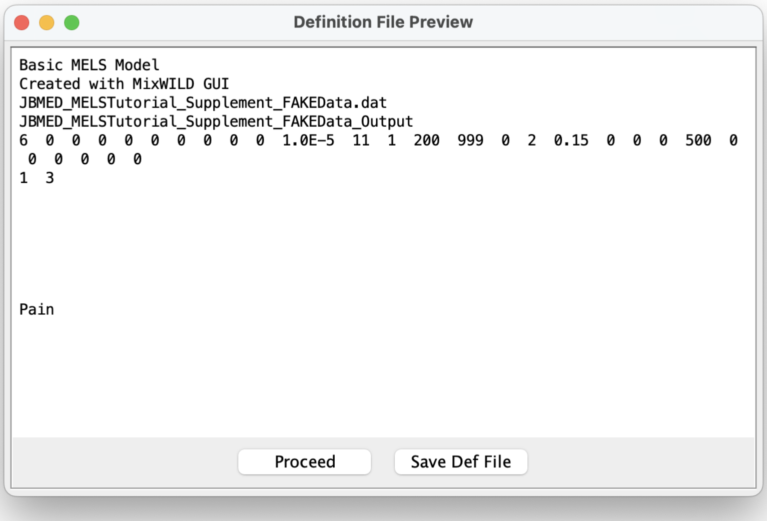
Definition File Preview
Basic MELS Model
Created with MixWILD GUI
JBMED_MELSTutorial_Supplement_FAKEData.dat
JBMED_MELSTutorial_Supplement_FAKEData_Output
6 0 0 0 0 0 0 0 0 0 1.0E-5 11 1 200 999 0 2 0.15 0 0 0 500 0
0 0 0 0 0
1 3
Pain
Proceed
Save Def File

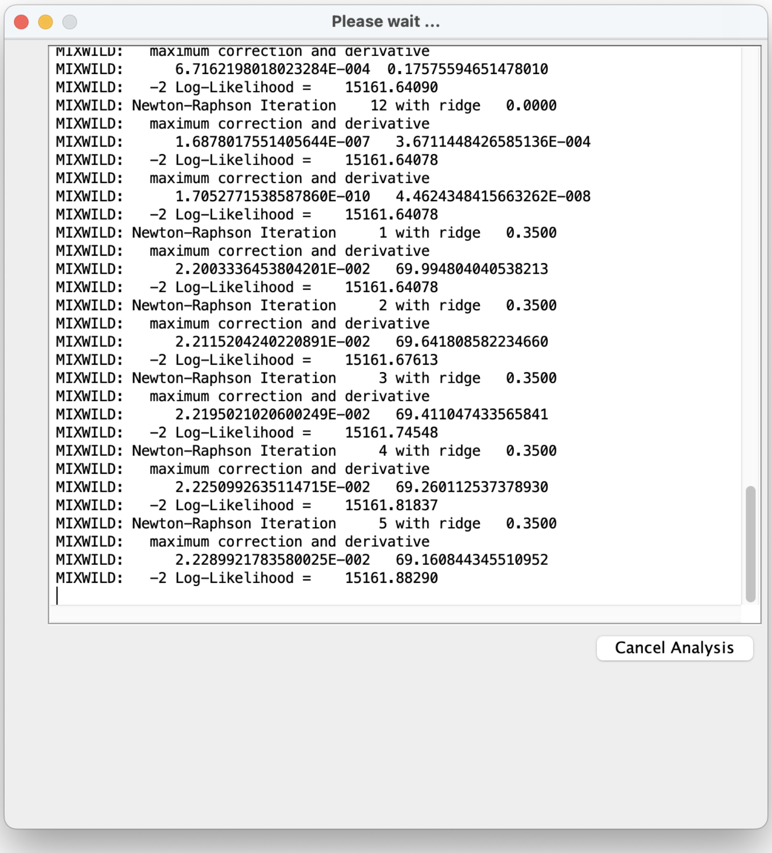

Finally, the results are produced, which can then be save as a file. The results from our analysis of the simulated data are provided as "1) Basic MELS Output.txt." Several sections can be found in the results, such as:

```
-----------------------------
Model without Scale Parameters
-----------------------------
```

If the model selected allows the residual variances to vary for each person, that is, "Include estimates of random scale." equals "Yes" from the first model selection screen, the desired results will be under the section "Model WITH RANDOM Scale." As all models in the paper allow the within-person variances to differ due to a random scale, the tables presented in the paper correspond to this section.

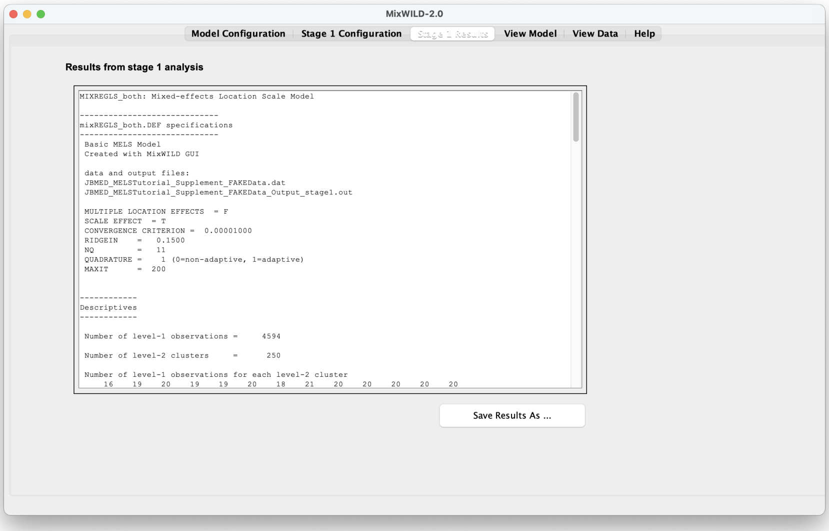

1.2 Model with Random Slopes

The procedure for controlling for random slopes is very similar to the Basic MELS model, with a few minor changes. Rather than reiterate all options, the following highlights differences in specifying the model relative to the basic model presented in the prior section.

On the first model specification page, as a random effect for time will be included (i.e., a random slope), the “Specify random location effects” is altered to include “Intercept and slope(s).”

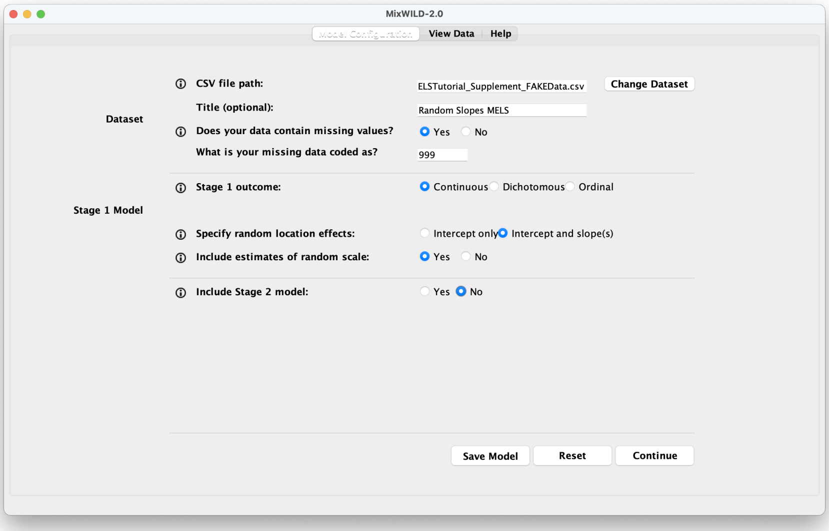

After hitting "Continue," the second model specification page appears a bit different, as the association of random location & scale is now no longer a user-specified choice. It is here that we "Configure Stage 1 Regressors" (left, below). This will introduce a new screen, "Add Stage 1 Regressors" (right, below). It is here that "Day," or whatever the index of time is named, is selected from the "Variables" list on the left and added to the "Level 1 (Time-Varying)" list on the right.

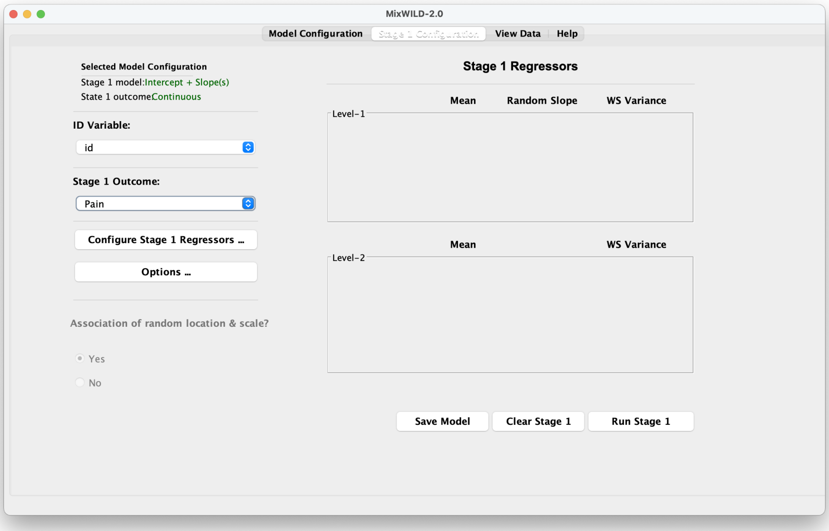

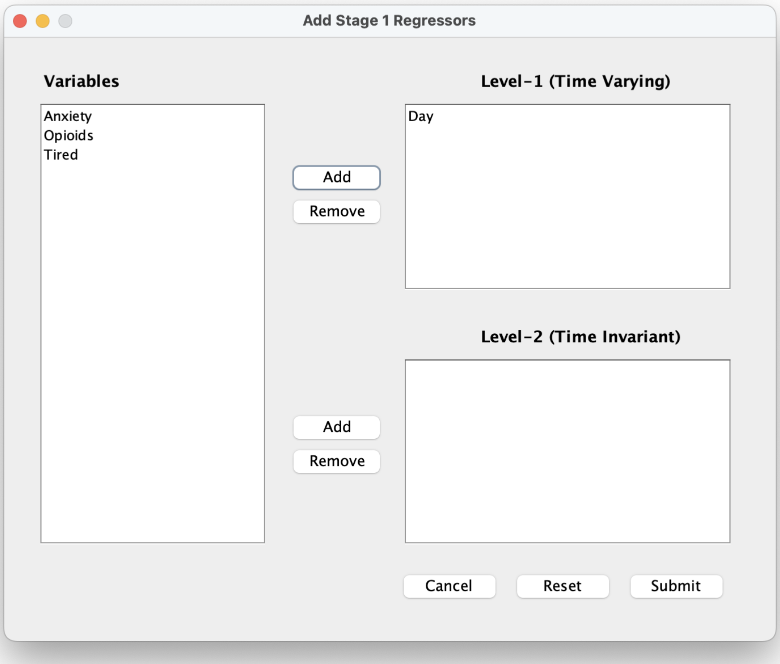

After submitting the Stage 1 Regressors, the earlier screen appears with new options. The Level-1 predictor "Day" can now be included in differing parts of the model. The checkbox under "mean" includes Day in the model as a fixed effect, allowing the average effect of "Day" across individuals to differ from zero. The checkbox under "Random Slope" includes a random effect for "Day," allowing individuals to have differing slopes associated with "Day." The check box under "WS Variance" is not selected in the paper example but would allow "Day" to predict changes in variance within people; if there was an expectation that the variance of residuals increases or decreases with study duration, this checkbox could be selected. Each of the checked boxes can also be disaggregated by selecting the corresponding "Disagregate?" checkbox. Disaggregation will divide time-varying regressors into two components: the mean of the regressor for each individual and how an individual's time-varying scores differ from their individual mean. This is a common choice in many longitudinal, multilevel models to disentangle between-subject and within-subject effects. This is less commonly applied to indices of time, but should be considered for other time-varying regressors, as in the final example.

Unlike the Basic MELS model, the option "Specify the relationship between mean and WS variance" (see page 4 for adidtional disucssion) has been replaced with "Association of random location & scale?" The reader will see that this option is grayed out, and has automatically been selected to be "Yes."

This allows there to be a covariance between the random effects specified in the location model (i.e., random intercept, random slope) and the within-subject variance expressed in the scale model. This allows for the possibility, for example, that individuals with steeper slopes may exhibit less variance around their trajectory. In contrast, those with a shallow slope may be primarily characterized by fluctuations and may consequently show higher variance. While this may not be the relation specifically expected in one's data, the "Yes" in this case allows the relation between random effects and the within-subject variance to differ from zero.

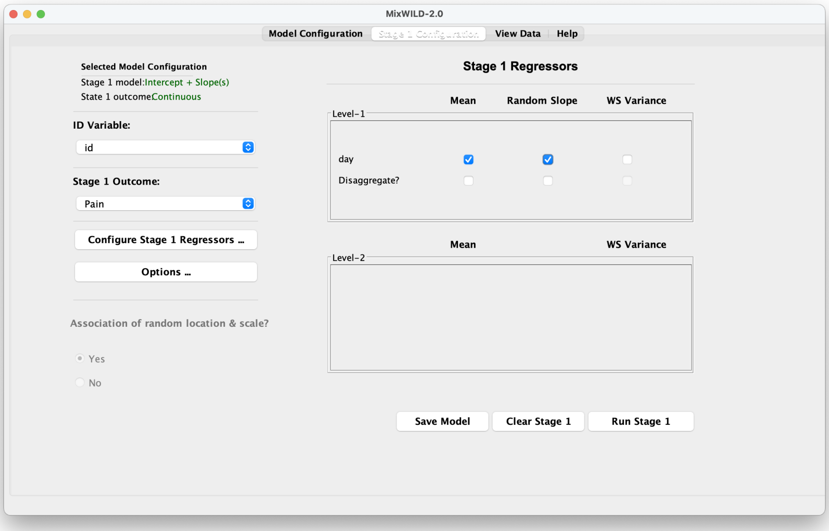

As with the Basic MELS model, one can now "Run Stage 1," "Proceed" through the analysis, and "Save Results As...." As before, the final section of the output, "Model WITH RANDOM Scale" includes the within-person differences of variance and corresponds to the tables presented in the paper. The results from our analysis of the simulated data are provided in "2) Random Slopes MELS Output.txt."

1.3 Modeling Within Subject Variance

In the final section, we consider modeling the within-subject variance, using first time-invariant predictors (Anxiety, Opioids) and then a time-varying predictor (Tired). The first screen for specifying the model is the same as the Basic MELS model, as is the initial second screen for specifying the model.

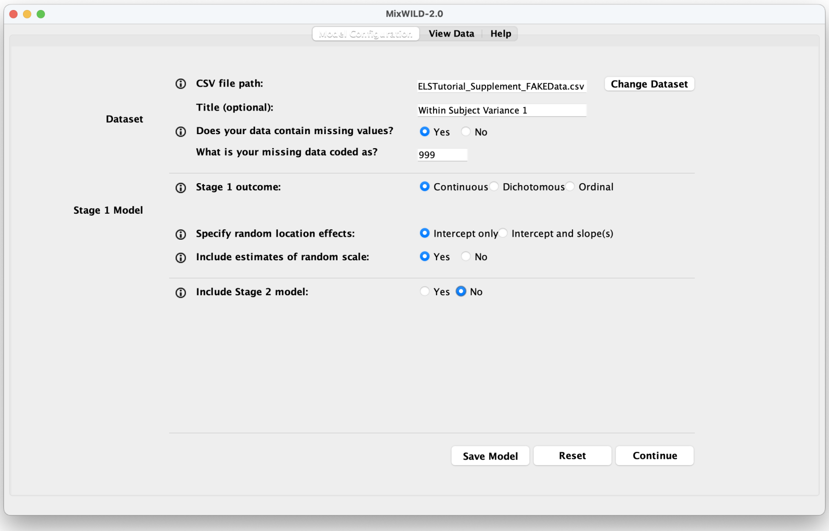

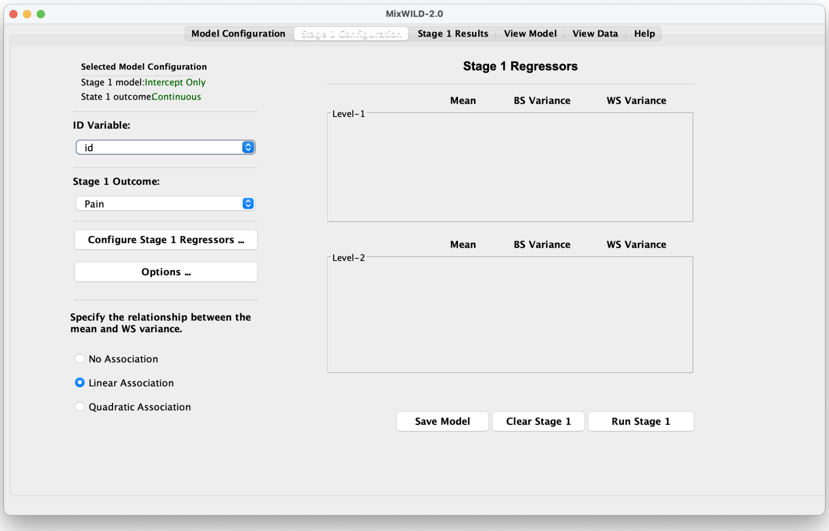

As with the random slopes, one must “Configure Stage 1 Regressors,” select “Anxiety” and “Opioids” from the “Variables” list, and add them as “Level-2 (Time Invariant)” predictors. These predictors are time-invariant, as each person has the same value for all observed occasions on these two variables.

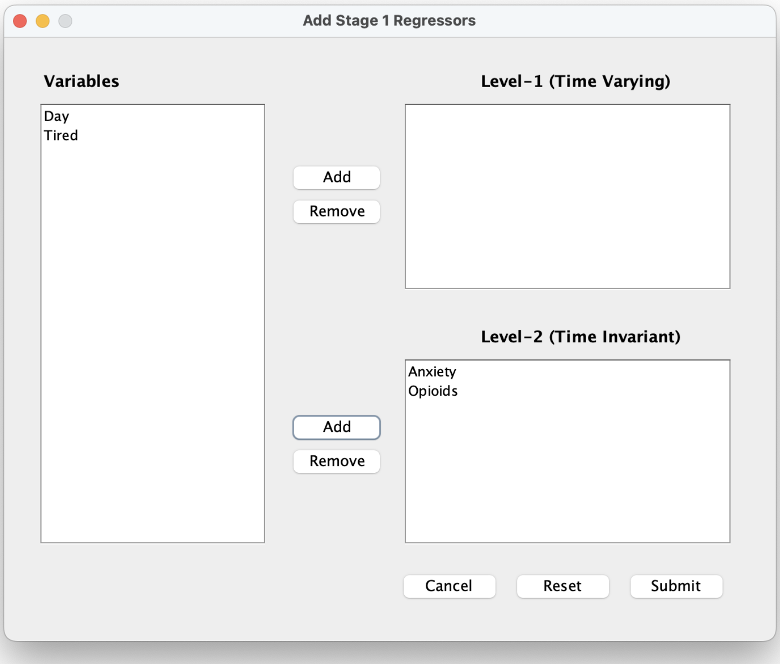

Submitting the stage 1 regressors results in the variables being listed in the "Level-2" box, as well as options as to how the regressors should be included in the model. Checking the boxes under "Mean" includes the regressors as fixed effects in the model, such that differences in an anxiety diagnosis or opioid use could be associated with higher or lower reports of pain. Checking the boxes under "BS variance" allows the between-subject variance to differ as a function of the regressors. In the pain example, it may be expected that individuals without an anxiety disorder diagnosis would show more similar mean pain scores than individuals with an anxiety disorder diagnosis; this would suggest a difference in the variance expected for the anxiety and non-anxiety groups, which can be modeled with the "BS variance" column of checkboxes. Finally, checking boxes under "WS Variance" allows the within-person variances to be systematically higher or lower depending on the value of the regressor.

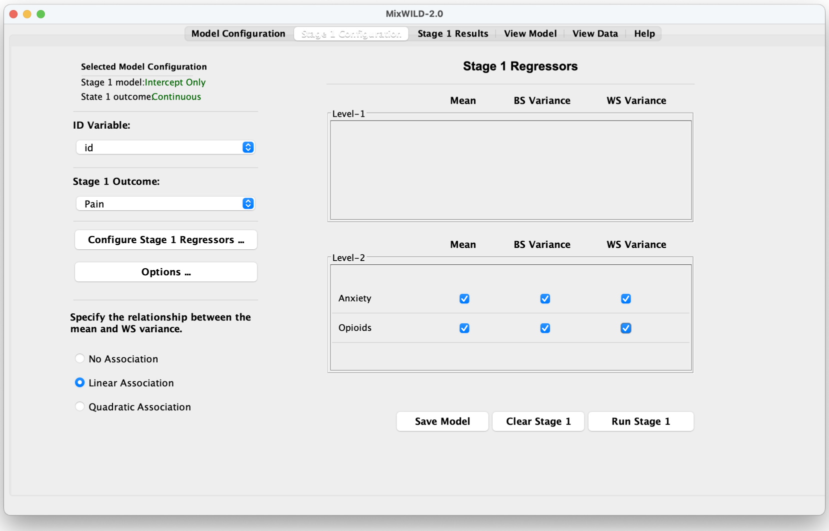

The remaining stages for running the model, saving output, and examining results are as in the prior sections. The results from our analysis of the simulated data are provided in "3) Within Subject Variance 1 Output.txt.'

The steps for a time-varying predictor are the same with the time-invariant predictors, except that when configuring the stage 1 regressors, the regressors are added under "Level-1 (Time Varying)."

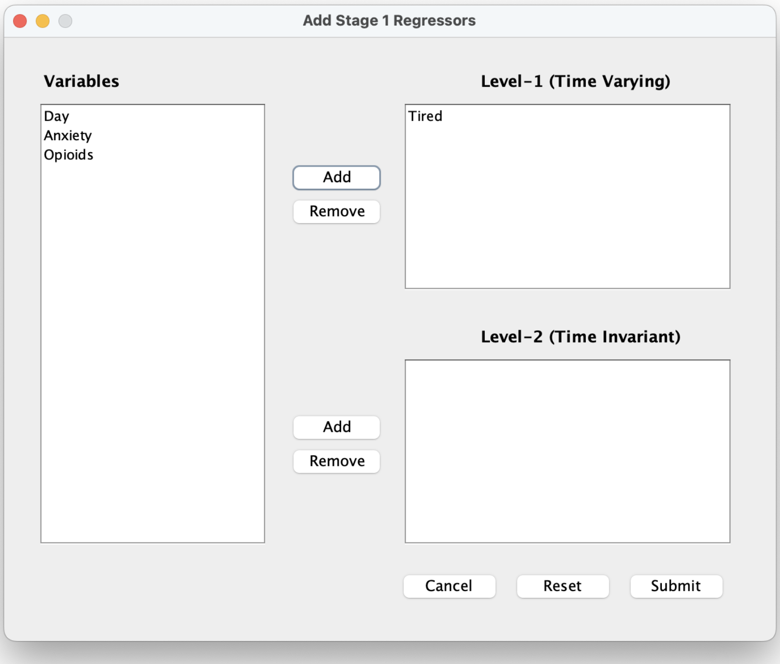

Like the addition of “Day” in the random slopes example, the time-varying regressor “Tired” will then appear in the “Level-1” box. Similar to the time-invariant example, the time-varying predictor can be included in the modeling of the “Mean,” the “BS Variance,” and the “WS Variance.” As highlighted when discussing the modeling of pain in the primary paper, the substantive interpretation of each of these differs substantially, and ideally, one would have substantive reasons for the selections made. The primary difference in this example is the checking of the disaggregation boxes, which will disaggregate the time-varying “Tired” predictor into between- and within-person differences, as is commonly done in multilevel models to disentangle effects.

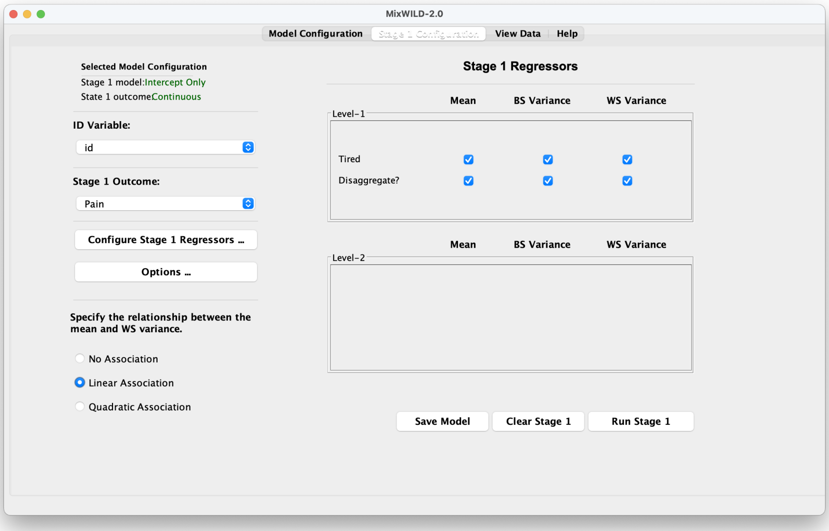

The remaining stages for running the model, saving output, and examining results are as in the prior sections. This includes the selection for "Specify the relationship between the means and WS variance," which is discussed in more detail on page 4. The results from our analysis of the simulated data are provided in "4) Within Subject Variance 2 Output.txt.'

MELS (brms version)

2025-08-19

2 brms package in R

This document outlines how to partially replicate the results of section 1 of this supplement using brms. Brms is an R package that can run Bayesian generalized multi-level models, with a similar syntax to familiar multi-level modeling packages like lme4. Brms provides an accessible interface to STAN (link), which is a flexible programming language for implementing Bayesian models (including MELS models). Currently, brms does not support modeling of between-variance components, but does support modeling within-individual variance components and thus can incorporate some scale effects. For between-individual variance modeling other software implementations are preferable (e.g., MixWiLD, MPlus, or coding directly in STAN). However, if between-individual variance modeling is not central to the research questions of interest brms provides an accessible option for users who prefer R over MixWILD. This supplement will demonstrate how to run brms models that parallel the models in the MixWILD supplement, and how the parameters in each software program line up, for substantive interpretation information, see the main text.

One note of caution, in the limited simulation testing we did comparing outputs from MixWILD and brms, we found that the estimates sometimes differed between MixWILD and brms (highlighted in the examples below). We were unable to resolve or determine the cause of these differences in estimates, but could not find a case where the statistical inferences differed. In our testing, MixWILD was able to more precisely recover estimates from simulated data. However, given the ubiquity of R and the general utility of brms, we felt that demonstrating how to implement MELS models within brms was still worthwhile.

```
rm(list=ls())
setwd("/uufs/chpc.utah.edu/common/home/u1041705/JBMED MELS")
```

The first step is to load necessary packages.

```
# uncomment following lines if using packages for the first time
#install.packages("brms")
#install.packages("tidyverse")
#install.packages("readr")

# LOAD NECESSARY PACKAGES
require(brms) #for bayesian MELS models
```

```
## Loading required package: brms

## Loading required package: Rcpp

## Loading 'brms' package (version 2.22.0). Useful instructions
## can be found by typing help('brms'). A more detailed introduction
## to the package is available through vignette('brms_overview').

##
## Attaching package: 'brms'

## The following object is masked from 'package:stats':
##
##     ar
```

```
require(tidyverse) #for data cleaning and centering
```

```
## Loading required package: tidyverse
## -- Attaching core tidyverse packages ------------------------ tidyverse 2.0.0 --
## v dplyr     1.1.4     v readr     2.1.5
## v forcats   1.0.0     v stringr   1.5.1
## v ggplot2   3.5.2     v tibble    3.3.0
## v lubridate 1.9.3     v tidyr     1.3.1
## v purrr     1.0.2
## -- Conflicts ------------------------------------------ tidyverse_conflicts() --
## x dplyr::filter() masks stats::filter()
## x dplyr::lag()    masks stats::lag()
## i Use the conflicted package (<http://conflicted.r-lib.org/>) to force all conflicts to become error
```

```
library(readr) #for read_csv() to load data
```

Next, load the data (available at https://osf.io/78jb3/)

```
# LOAD DATA
JBMED_MELS_SUPPLEMENT_DATA <- read_csv("JBMED_MELSTutorial_Supplement_FAKEData (1).csv")
```

```
## Rows: 5250 Columns: 6
## -- Column specification --------------------------------------------------------
## Delimiter: ","
## dbl (6): id, Day, Pain, Anxiety, Opioids, Tired
##
## i Use `spec()` to retrieve the full column specification for this data.
## i Specify the column types or set `show_col_types = FALSE` to quiet this message.
```

```
JBMED_MELS_SUPPLEMENT_DATA[JBMED_MELS_SUPPLEMENT_DATA == 999] <- NA
```

2.1 Basic MELS

The following code chunk runs a similar model to the basic MELS model presented in Section 3 of the paper.

```
# BASIC MELS (SECTION 2.1)
library(brms) #load brms
# brms equation
#specify id as grouping variable for both Pain (outcome) and within-individual variance (sigma)
mod1 <- bf(Pain ~ (1|c|id), sigma ~ (1|c|id)) # z specifies correlation between

# run brms model (model 1)
fit_model1 <- brm(mod1,
                       data = JBMED_MELS_SUPPLEMENT_DATA,
                       prior = NULL, #uniformative prior can specify otherwise
                       iter = 5000, #can increase if needed to solve convergence
                       save_pars = save_pars(all = TRUE),
                       refresh = 0) #supress chain updating output
```

```
## Running /uufs/chpc.utah.edu/sys/installdir/r8/R/4.4.2/lib64/R/bin/R CMD SHLIB \
##   foo.c
## using C compiler: ‘gcc (Spack GCC) 13.1.0’
## gcc -I"/uufs/chpc.utah.edu/sys/installdir/r8/R/4.4.2/lib64/R/include" -DNDEBUG   -I"/uufs/chpc.utah.
## In file included from /uufs/chpc.utah.edu/sys/installdir/r8/RLibs/4.4.2/RcppEigen/include/Eigen/Core
```

```
##                   from /uufs/chpc.utah.edu/sys/installdir/r8/RLibs/4.4.2/RcppEigen/include/Eigen/Dense
##                   from /uufs/chpc.utah.edu/common/home/u1041705/R/x86_64-pc-linux-gnu-library/4.4/Stan
##                   from <command-line>:
## /uufs/chpc.utah.edu/sys/installdir/r8/RLibs/4.4.2/RcppEigen/include/Eigen/src/Core/util/Macros.h:679
##   679 | #include <cmath>
##       |          ^~~~~~~
## compilation terminated.
## make: *** [/uufs/chpc.utah.edu/sys/installdir/r8/R/4.4.2/lib64/R/etc/Makeconf:195: foo.o] Error 1
```

```
summary(fit_model1) # model 1 summary
```

```
##  Family: gaussian
##   Links: mu = identity; sigma = log
## Formula: Pain ~ (1 | c | id)
##          sigma ~ (1 | c | id)
##    Data: JBMED_MELS_SUPPLEMENT_DATA (Number of observations: 4546)
##   Draws: 4 chains, each with iter = 5000; warmup = 2500; thin = 1;
##          total post-warmup draws = 10000
##
## Multilevel Hyperparameters:
## ~id (Number of levels: 250)
##                                 Estimate Est.Error l-95% CI u-95% CI Rhat
## sd(Intercept)                       0.22      0.03     0.16     0.27 1.00
## sd(sigma_Intercept)                 0.11      0.02     0.07     0.14 1.00
## cor(Intercept,sigma_Intercept)      0.26      0.19    -0.12     0.63 1.00
##                                 Bulk_ESS Tail_ESS
## sd(Intercept)                       3787     5496
## sd(sigma_Intercept)                 3074     3797
## cor(Intercept,sigma_Intercept)      2297     3067
##
## Regression Coefficients:
##                 Estimate Est.Error l-95% CI u-95% CI Rhat Bulk_ESS Tail_ESS
## Intercept           3.17      0.02     3.13     3.22 1.00     7813     7829
## sigma_Intercept     0.16      0.01     0.13     0.19 1.00     8620     7102
##
## Draws were sampled using sampling(NUTS). For each parameter, Bulk_ESS
## and Tail_ESS are effective sample size measures, and Rhat is the potential
## scale reduction factor on split chains (at convergence, Rhat = 1).
```

Under "Regression Coefficients," the "intercept" value (3.17) is the mean pain score across all observations and individuals, which lines up with the "intercept" value under the "BETA (regression coefficients)" heading in MixWILD. The "sigma_Intercept" value from brms (.16), which should theoretically line up to the "intercept" value under the "TAU (WS variance parameters:log-linear model)" heading in MixWILD.

Under "Multilevel Hyperparameters" the "sd(Intercept)" value (.22) is the estimated between individual variance, which lines up with the "intercept" value under "ALPHA (BS variance parameters: log-linear model)" in MixWILD (once you exponentiate and convert to standard deviation units (sqrt(exp(-3.05))=.22). Next, the "sd(sigma_Intercept)" is .11, and should theoretically line up with the "Std Dev" value under "Random scale standard deviation" in MixWILD. Finally, "cor(Intercept, sigma_Intercept) is .26, which represents something similar to the "Loc Eff" under "Random location (mean) effect of WS variance" in MixWILD, though the brms value is a direct correlation, while the MixWILD value is a regression parameter.

As noted at the begining of this supplement, there is a difference in estimates here for the "sigma_Intercept," and "sd(sigma_Intercept)" values between brms and MixWILD, in which the scaling seems to be consistently off by a factor of around 2 for certain values in the brms output (.16 vs. .32 and .11 vs. .21 respectively in brms vs. MixWild). In comparing the credible intervals from brms to the z/p-values from MixWILD, the

inferences are similar despite this scaling difference.

2.2 Model with Random Slopes

```
# RANDOM SLOPES (SECTION 2.2)
# model 2 brms equation
mod2 <- bf(Pain ~ Day + (1 + Day|c|id), sigma ~ (1|c|id))

# run brms model (model 2)
fit_model2 <- brm(mod2,
                       data = JBMED_MELS_SUPPLEMENT_DATA,
                       prior = NULL, #uniformative prior can specify otherwise
                       iter = 5000, #can increase if needed to solve convergence
                       save_pars = save_pars(all = TRUE),
                       refresh = 0) #supress chain updating output
```

```
## Warning: Rows containing NAs were excluded from the model.

## Compiling Stan program...

## Trying to compile a simple C file

## Running /uufs/chpc.utah.edu/sys/installdir/r8/R/4.4.2/lib64/R/bin/R CMD SHLIB \
##   foo.c
## using C compiler: ‘gcc (Spack GCC) 13.1.0’
## gcc -I"/uufs/chpc.utah.edu/sys/installdir/r8/R/4.4.2/lib64/R/include" -DNDEBUG   -I"/uufs/chpc.utah.
## In file included from /uufs/chpc.utah.edu/sys/installdir/r8/RLibs/4.4.2/RcppEigen/include/Eigen/Core
##                  from /uufs/chpc.utah.edu/sys/installdir/r8/RLibs/4.4.2/RcppEigen/include/Eigen/Dens
##                  from /uufs/chpc.utah.edu/common/home/u1041705/R/x86_64-pc-linux-gnu-library/4.4/Sta
##                  from <command-line>:
## /uufs/chpc.utah.edu/sys/installdir/r8/RLibs/4.4.2/RcppEigen/include/Eigen/src/Core/util/Macros.h:679
##   679 | #include <cmath>
##       |          ^~~~~~~
## compilation terminated.
## make: *** [/uufs/chpc.utah.edu/sys/installdir/r8/R/4.4.2/lib64/R/etc/Makeconf:195: foo.o] Error 1

## Start sampling
```

```
summary(fit_model2) # model 2 summary
```

```
##  Family: gaussian
##   Links: mu = identity; sigma = log
## Formula: Pain ~ Day + (1 + Day | c | id)
##          sigma ~ (1 | c | id)
##    Data: JBMED_MELS_SUPPLEMENT_DATA (Number of observations: 4546)
##   Draws: 4 chains, each with iter = 5000; warmup = 2500; thin = 1;
##          total post-warmup draws = 10000
##
## Multilevel Hyperparameters:
## ~id (Number of levels: 250)
##                                  Estimate Est.Error l-95% CI u-95% CI Rhat
## sd(Intercept)                        0.22      0.06     0.08     0.34 1.00
## sd(Day)                              0.05      0.01     0.02     0.08 1.00
## sd(sigma_Intercept)                  0.12      0.02     0.08     0.15 1.00
## cor(Intercept,Day)                  -0.34      0.34    -0.78     0.55 1.00
```

```
## cor(Intercept,sigma_Intercept)     0.64      0.22     0.14     0.96 1.01
## cor(Day,sigma_Intercept)          -0.51      0.25    -0.91     0.06 1.00
##                                 Bulk_ESS Tail_ESS
## sd(Intercept)                       2721     2777
## sd(Day)                              700     1118
## sd(sigma_Intercept)                 4557     6170
## cor(Intercept,Day)                  1184     1577
## cor(Intercept,sigma_Intercept)       811     1500
## cor(Day,sigma_Intercept)            1155     1608
##
## Regression Coefficients:
##                 Estimate Est.Error l-95% CI u-95% CI Rhat Bulk_ESS Tail_ESS
## Intercept           3.13      0.04     3.05     3.22 1.00    14332     8064
## sigma_Intercept     0.16      0.01     0.13     0.18 1.00     9215     8012
## Day                 0.01      0.01    -0.01     0.03 1.00    14567     7907
##
## Draws were sampled using sampling(NUTS). For each parameter, Bulk_ESS
## and Tail_ESS are effective sample size measures, and Rhat is the potential
## scale reduction factor on split chains (at convergence, Rhat = 1).
```

For the random slopes model, under “Regression Coefficients,” the “intercept” value (3.13) is the mean pain score across all individuals when “Day” is 0, which lines up with the “intercept” value under the “BETA (regression coefficients)” in MixWILD. The “sigma_Intercept” value from brms (.16) should theoretically line up to the “intercept” value under “TAU (WS variance parameters:log-linear model)” in MixWILD. The “Day” value (.01) in brms is the estimate for change in pain for each additional day, which lines up with the “Day” value under “BETA (regression coefficients)” in MixWILD.

Under “Multilevel Hyperparameters,” the “sd(Intercept)” (.22) and “sd(Day)” (.05) values in brms should line up with respectively with the “intercept” and “Day” values under “Random (location) Effect Variances and Covariances” in MixWILD.

The brms “sd(sigma_Intercept)” (.12) value should line up with “scale int var” under “Random scale Variance and Covariance” in MixWILD. For the correlation values listed on the brms output, if converted to covariances, “cor(Intercept,Day)” (-.34) should line up with the “Covariance” value under “Random (location) Effect Variances and Covariances”, “cor(Intercept,sigma_Intercept)” (.64) should line up with “Cov intercept”, and “cor(Day,sigma_Intercept)” (-.51) should line up with “Cov Day” in MixWILD. Again, the output numbers do not exactly line up here, in our assessement this may be due to differences in how the model output is formatted (covariance/variance vs. correlation/standard deviation) or the aforementioned scaling issue.

2.3 Modeling within-individual variance

2.31 Time-invariant predictors (Anxiety, Opioid use) of within-individual variance

```r
# SECTION 5.1
# model 3 brms equation
#Anxiety and Opioids as predictors of Pain and within-individual variance (sigma)
mod3 <- bf(Pain ~ Anxiety + Opioids + (1|c|id), sigma ~  Anxiety + Opioids + (1|c|id))

# run brms model (model 3)
fit_model3 <- brm(mod3,
                  data = JBMED_MELS_SUPPLEMENT_DATA,
                  prior = NULL, #uniformative prior can specify otherwise
```

```r
                        iter = 5000, #can increase if needed to solve convergence
                        save_pars = save_pars(all = TRUE),
                        refresh = 0) #supress chain updating output
```

```
## Warning: Rows containing NAs were excluded from the model.

## Compiling Stan program...

## Trying to compile a simple C file

## Running /uufs/chpc.utah.edu/sys/installdir/r8/R/4.4.2/lib64/R/bin/R CMD SHLIB \
##   foo.c
## using C compiler: ‘gcc (Spack GCC) 13.1.0’
## gcc -I"/uufs/chpc.utah.edu/sys/installdir/r8/R/4.4.2/lib64/R/include" -DNDEBUG   -I"/uufs/chpc.utah.
## In file included from /uufs/chpc.utah.edu/sys/installdir/r8/RLibs/4.4.2/RcppEigen/include/Eigen/Core
##                  from /uufs/chpc.utah.edu/sys/installdir/r8/RLibs/4.4.2/RcppEigen/include/Eigen/Dens
##                  from /uufs/chpc.utah.edu/common/home/u1041705/R/x86_64-pc-linux-gnu-library/4.4/Sta
##                  from <command-line>:
## /uufs/chpc.utah.edu/sys/installdir/r8/RLibs/4.4.2/RcppEigen/include/Eigen/src/Core/util/Macros.h:679
##   679 | #include <cmath>
##       |          ^~~~~~~
## compilation terminated.
## make: *** [/uufs/chpc.utah.edu/sys/installdir/r8/R/4.4.2/lib64/R/etc/Makeconf:195: foo.o] Error 1

## Start sampling
```

```r
summary(fit_model3) #model 3 summary
```

```
##  Family: gaussian
##   Links: mu = identity; sigma = log
## Formula: Pain ~ Anxiety + Opioids + (1 | c | id)
##          sigma ~ Anxiety + Opioids + (1 | c | id)
##    Data: JBMED_MELS_SUPPLEMENT_DATA (Number of observations: 4546)
##   Draws: 4 chains, each with iter = 5000; warmup = 2500; thin = 1;
##          total post-warmup draws = 10000
##
## Multilevel Hyperparameters:
## ~id (Number of levels: 250)
##                                Estimate Est.Error l-95% CI u-95% CI Rhat
## sd(Intercept)                      0.19      0.03     0.13     0.24 1.00
## sd(sigma_Intercept)                0.11      0.02     0.07     0.14 1.00
## cor(Intercept,sigma_Intercept)     0.21      0.21    -0.21     0.63 1.00
##                                Bulk_ESS Tail_ESS
## sd(Intercept)                      3348     4191
## sd(sigma_Intercept)                3432     5125
## cor(Intercept,sigma_Intercept)     2327     3112
##
## Regression Coefficients:
##                 Estimate Est.Error l-95% CI u-95% CI Rhat Bulk_ESS Tail_ESS
## Intercept           3.03      0.03     2.96     3.09 1.00     9826     7419
## sigma_Intercept     0.14      0.02     0.10     0.18 1.00     9474     7853
## Anxiety             0.20      0.04     0.12     0.28 1.00     9625     7802
## Opioids             0.14      0.04     0.06     0.23 1.00    10784     7898
## sigma_Anxiety       0.03      0.03    -0.02     0.08 1.00    10470     7325
## sigma_Opioids       0.03      0.03    -0.03     0.08 1.00    10757     7896
##
```

```
## Draws were sampled using sampling(NUTS). For each parameter, Bulk_ESS
## and Tail_ESS are effective sample size measures, and Rhat is the potential
## scale reduction factor on split chains (at convergence, Rhat = 1).
```

Under the "Regression Coefficients" heading in brms, "Intercept" (3.03), "Anxiety" (.20), and "Opioids" (.14), lines up with respectively with "intercept", "Anxiety, and"Opioid" values under "BETA (regression coefficents)" in MixWILD. Next, "sigma_Intercept" (.14), "sigma_Anxiety"(.03), and "sigma_Opiods"(.03), should line up respectively with "intercept", "Anxiety", and "Opioids" under "TAU (WS variance parameters: log-linear model)" in MixWILD (though we see the scaling issue with the brms values being 1/2 the MixWILD values).

As a reminder, brms does not currently allow for the modeling of the between-individual variance component, thus everything under the "ALPHA (BS variance parameters: log-linear model)" in MixWILD is collapsed to "sd(Intercept)" (.19) in brms under "Multilevel Hyperparameters". Under this same heading, "sd(sigma_Intercept)" (.11) should line up with "Std Dev" under "Random scale standard deviation" in MixWILD (scaling difference again here). Finally, "cor(Intercept,sigma_Intercept)" (.21) represents something similar to the "Loc Eff" under "Random location (mean) effect of WS variance" in MixWILD, though as mentioned before the values are presented in different formats (correlation vs. regression parameter).

2.32 Time-varying predictor (Fatigue/Tired) of within-individual variance

```
# SECTION 5.2
# centering
JBMED_MELS_SUPPLEMENT_DATA <- JBMED_MELS_SUPPLEMENT_DATA %>%
group_by(id) %>%
mutate(Tired_im = mean(Tired, na.rm=TRUE)) %>%
ungroup() %>%
mutate(Tired_WS = Tired - Tired_im,
       Tired_BS = Tired_im)

# model 4 brms equation
mod4 <- bf(Pain ~ Tired_BS + Tired_WS +  (1|c|id), sigma ~ Tired_BS + Tired_WS + (1|c|id))

# run brms model (model 4)
fit_model4 <- brm(mod4,

                       data = JBMED_MELS_SUPPLEMENT_DATA,
                       prior = NULL, #uniformative prior can specify otherwise
                       iter = 5000, #can increase if needed to solve convergence
                       save_pars = save_pars(all = TRUE),
                       refresh = 0) #supress chain updating output
```

```
## Warning: Rows containing NAs were excluded from the model.

## Compiling Stan program...

## Trying to compile a simple C file

## Running /uufs/chpc.utah.edu/sys/installdir/r8/R/4.4.2/lib64/R/bin/R CMD SHLIB \
##   foo.c
## using C compiler: ‘gcc (Spack GCC) 13.1.0’
## gcc -I"/uufs/chpc.utah.edu/sys/installdir/r8/R/4.4.2/lib64/R/include" -DNDEBUG   -I"/uufs/chpc.utah.
## In file included from /uufs/chpc.utah.edu/sys/installdir/r8/RLibs/4.4.2/RcppEigen/include/Eigen/Core
##                  from /uufs/chpc.utah.edu/sys/installdir/r8/RLibs/4.4.2/RcppEigen/include/Eigen/Dens
##                  from /uufs/chpc.utah.edu/common/home/u1041705/R/x86_64-pc-linux-gnu-library/4.4/Sta
##                  from <command-line>:
```

```
## /uufs/chpc.utah.edu/sys/installdir/r8/RLibs/4.4.2/RcppEigen/include/Eigen/src/Core/util/Macros.h:679
##   679 | #include <cmath>
##       |          ^~~~~~~
## compilation terminated.
## make: *** [/uufs/chpc.utah.edu/sys/installdir/r8/R/4.4.2/lib64/R/etc/Makeconf:195: foo.o] Error 1

## Start sampling

## Warning: Bulk Effective Samples Size (ESS) is too low, indicating posterior means and medians may be
## Running the chains for more iterations may help. See
## https://mc-stan.org/misc/warnings.html#bulk-ess

## Warning: Tail Effective Samples Size (ESS) is too low, indicating posterior variances and tail quant
## Running the chains for more iterations may help. See
## https://mc-stan.org/misc/warnings.html#tail-ess
```

```
summary(fit_model4) #model 4 summary
```

```
##  Family: gaussian
##   Links: mu = identity; sigma = log
## Formula: Pain ~ Tired_BS + Tired_WS + (1 | c | id)
##          sigma ~ Tired_BS + Tired_WS + (1 | c | id)
##    Data: JBMED_MELS_SUPPLEMENT_DATA (Number of observations: 4546)
##   Draws: 4 chains, each with iter = 5000; warmup = 2500; thin = 1;
##          total post-warmup draws = 10000
##
## Multilevel Hyperparameters:
## ~id (Number of levels: 250)
##                                Estimate Est.Error l-95% CI u-95% CI Rhat
## sd(Intercept)                      0.12      0.04     0.02     0.18 1.01
## sd(sigma_Intercept)                0.16      0.02     0.13     0.19 1.00
## cor(Intercept,sigma_Intercept)     0.18      0.27    -0.40     0.69 1.01
##                                Bulk_ESS Tail_ESS
## sd(Intercept)                       618      247
## sd(sigma_Intercept)                4453     7119
## cor(Intercept,sigma_Intercept)      397      274
##
## Regression Coefficients:
##                 Estimate Est.Error l-95% CI u-95% CI Rhat Bulk_ESS Tail_ESS
## Intercept           0.47      0.23     0.03     0.91 1.00    11927     8116
## sigma_Intercept    -0.14      0.19    -0.51     0.24 1.00     8382     7739
## Tired_BS            0.69      0.06     0.57     0.80 1.00    11966     8147
## Tired_WS            0.35      0.01     0.33     0.38 1.00    15384     6741
## sigma_Tired_BS      0.05      0.05    -0.04     0.15 1.00     8399     7741
## sigma_Tired_WS      0.02      0.01     0.01     0.04 1.00    16525     7861
##
## Draws were sampled using sampling(NUTS). For each parameter, Bulk_ESS
## and Tail_ESS are effective sample size measures, and Rhat is the potential
## scale reduction factor on split chains (at convergence, Rhat = 1).
```

Under the "Regression Coefficients" heading in brms, "Intercept" (.47), "Tired_BS" (.69), and "Tired_WS" (.35), lines up with respectively with "intercept", Tired_BS", and "Tired_WS" under "BETA (regression coefficents)" in MixWILD. Next, "sigma_Intercept" (-.14), "sigma_Tired_BS"(.05), and "sigma_Tired_WS"(.02), should line up respectively with "intercept", Tired_BS", and "Tired_WS" under "TAU (WS variance parameters: log-linear model)" in MixWILD (setting aside the scaling difference again here).

Moving to "Multilevel Hyperparameters" in brms. As in the prior model, everything under the "ALPHA

(BS variance parameters: log-linear model)” in MixWILD collapses under “sd(Intercept)” (.12) in brms. As before, “sd(sigma_Intercept)” (.16) should line up with “Std Dev” under “Random scale standard deviation” in MixWILD (scaling difference again here) and “cor(Intercept,sigma_Intercept)” (.18) represents something similar to the “Loc Eff” under “Random location (mean) effect of WS variance” in MixWILD, though as mentioned before the values are presented in different formats (correlation vs. regression parameter).

2 Checking Convergence

As with the modeling of any data, users should be aware that convergence or the appearance of results does not in itself ensure that the models have converged on a reasonable solution. Given the complexity of the MELS models that can be specified, the possibility of empirical underidentification should be taken into account. Like other methods, careful examination of the output can often provide warning signs that the estimates should not be trusted. In generating fake data for this supplement, the authors produced a model that converged; however, multiple indicators suggested that the results should not be trusted. A figure of this output is shown below. As with other statistical methods, excessively large estimates or estimates that lend themselves to unreasonable inferences, such as those in the blue box, are one red flag. Another red flag is missing or unusually large estimates for the standard errors, as in the red box. Finally, the variances and covariances in data are rarely exactly equal to zero, and consequently, the pink box also highlights a red flag. The presence of any one of these red flags should lead to a re-evaluation of whether the data are sufficient to support the complexity of the model being considered, and whether the inferences can be trusted.

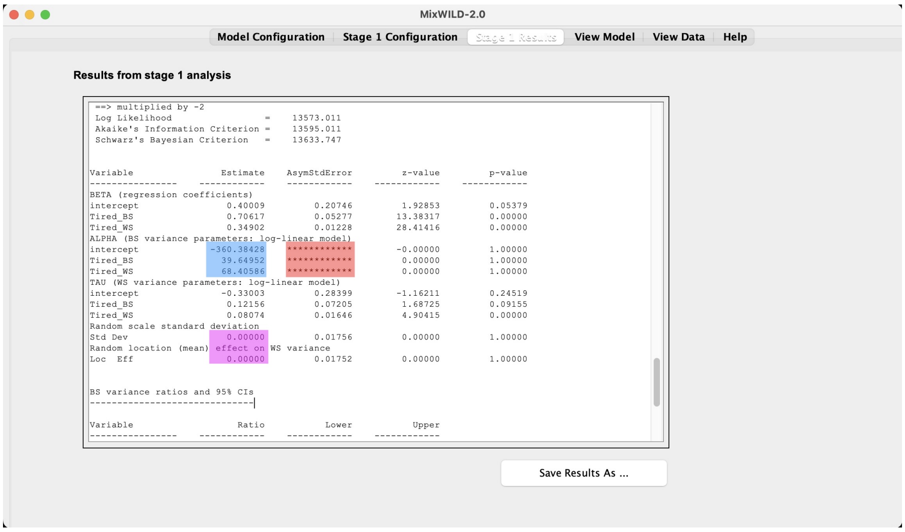

Supplement: Supplementary file 1 — (pdf 6023 KB) [file 10865_2026_638_MOESM1_ESM.pdf]
